# Microbiota‐Targeted Chitooligosaccharides Intervention Restores Glucose Homeostasis After Islet Cell Transplantation in Rapamycin‐Treated Mice

**DOI:** 10.1002/fsn3.72122

**Published:** 2026-07-17

**Authors:** Kunlin Chang, Duowen He, Junfeng Dong, Yayu Zhang, Shuxin Deng, Mengyao Zhao, Jiayang Jin, Xiaoguo Ji, Hao Yin, Liming Zhao

**Affiliations:** ^1^ State Key Laboratory of Bioreactor Engineering, School of Biotechnology East China University of Science and Technology Shanghai China; ^2^ Organ Transplant Center Shanghai Changzheng Hospital (Second Affiliated Hospital of Naval Medical University) Shanghai China; ^3^ Shanghai Frontiers Science Center of Optogenetic Techniques for Cell Metabolism Shanghai China; ^4^ Shanghai Collaborative Innovation Center for Biomanufacturing Technology (SCICBT) Shanghai China; ^5^ Medical‐Engineering Integration Innovation Center East China University of Science and Technology Shanghai China

**Keywords:** chitooligosaccharides, glucose homeostasis, gut microbiota, islet cell transplantation, rapamycin

## Abstract

Islet cell transplantation (ICT) is an effective treatment for diabetes mellitus, but postoperative islet function recovery and inflammation are closely linked to immunosuppressants. Using multi‐omics and fecal microbiota transplantation (FMT) in human microbiota‐associated (HMA) mice, this study explored rapamycin‐induced gut dysbiosis and its impacts on islet function and inflammation post‐ICT. ICT significantly altered the gut microbiota of type 2 diabetes mellitus (T2DM) patients, and FMT from these patients to antibiotic‐treated mice recapitulated metabolic disorders in the mice. These disorders included hyperglycemia, hepatic and pancreatic injury, and impaired intestinal barrier. Rapamycin decreased beneficial bacteria (*Akkermansia*, *Faecalibacterium*) and enriched *Desulfovibrio* in HMA‐T2DM mice. Targeted microbial modulation by chitooligosaccharides (COS) ameliorated rapamycin‐induced deficits in insulin and C‐peptide secretion, as well as elevated glycated hemoglobin levels. COS also significantly reduced serum inflammatory markers IP‐10 and MCP‐1, while upregulating colonic barrier proteins (*Muc2*, *Occludin*) in HMA‐T2DM‐ICT mice. COS additionally mitigated postoperative hyperglycemia via the PI3K/AKT/GSK3β/FOXO1 signaling pathway. This study identified COS as a microbiota‐targeted adjunctive strategy to improve metabolic recovery and islet function under post‐transplant immunosuppression.

## Graphical abstract

Gut microbiota from islet cell transplantation patients treated with rapamycin significantly elevated blood glucose levels in recipient mice. Chitooligosaccharides improved rapamycin‐induced gut dysbiosis and islet dysfunction in human microbiota‐associated T2DM mice. Chitooligosaccharides restored islet function and reduced inflammation via the PI3K/AKT/GSK3β/FOXO1 pathway and modulation of gut microbiota.

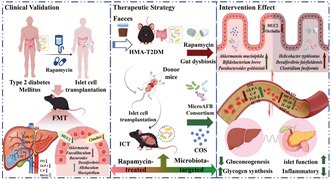

## 1 Introduction

Islet cell transplantation (ICT) is a minimally invasive therapy involving the isolation, purification, and interventional infusion of pancreatic islet cells from allogeneic or autologous donors (Verhoeff et al. 2021). Transplanted islets secrete insulin physiologically, stabilizing blood glucose, alleviating hypoglycemia, protecting systemic organs and vessels, and preventing diabetic end‐stage complications. ICT represents an effective therapy for T2DM, and some patients achieve long‐term insulin independence with markedly improved quality of life (Triolo and Bellin 2021). However, post‐transplant decline in islet function, suboptimal long‐term efficacy, and significant glycemic variability remain major challenges for ICT (Salama et al. 2024). The sustainability of islet function and stability of glycemic control post‐transplantation were limited by multiple factors (Czarnecka et al. 2023). Importantly, long‐term immunosuppressant and antibiotic treatment (e.g., tacrolimus and rapamycin) induce severe gut dysbiosis marked by reduced microbial diversity, depleted commensal bacteria and expanded opportunistic pathogens (Mohamed et al. 2024). This microbial imbalance damaged intestinal barrier integrity to trigger bacterial translocation and systemic mild inflammation, and altered host immunity to compromise graft islet survival and function (Gabarre et al. 2022). Therefore, restoring gut microbial homeostasis may serve as a promising strategy to ameliorate immunosuppression‐related gut injury and improve the long‐term survival of transplanted islets.

Rapamycin and tacrolimus contribute to gut dysbiosis, whereas gut microbiota homeostasis is critical for systemic energy metabolism and immune regulation (Gabarre et al. 2025). Following renal transplantation, recipients frequently develop severe diarrhea or recurrent urinary tract infections attributable to prolonged tacrolimus exposure (Hu et al. 2025). Calcineurin inhibitors can directly disrupt tight junctions between enterocytes, increasing intestinal permeability and driving the translocation of commensals and their metabolites, which exacerbates dysbiosis (Kunasol et al. 2025). Therefore, we hypothesized that impaired islet function recovery and post‐transplant glycemic fluctuations were associated with gut microbial dysbiosis induced by long‐term administration of rapamycin.

Gut microbiota imbalance has been recognized as a critical modulator of glycemic regulation and islet physiology, and it is an important therapeutic target for alleviating adverse reactions related to immunosuppression and promoting the recovery of host functions (Faucher et al. 2022). As core prebiotics, functional oligosaccharides reach the colon intact, enrich beneficial microbiota (e.g., *Bifidobacterium*, *Lactobacillus*), inhibit pathogen overgrowth, and synergistically regulate glucose metabolism and exert anti‐inflammatory effects (Dimba et al. 2024). Oral administration of Astragalus polysaccharides (Rong and Shu 2024) and *Holothuria leucospilota* polysaccharides (Zhao et al. 2023) targeted modulate butyrate‐producing gut microbiota and enhanced the immunoregulatory capacity of cyclophosphamide‐induced immunosuppressed mice. Among these bioactive carbohydrates, chitooligosaccharides (COS) have been reported to regulate macrophage polarization through the MyD88/NF‐κB‐STAT6 axis, attenuate post‐transplant islet inflammation, and thus constitute a promising adjunctive intervention for optimizing ICT outcomes (Zhang, Ji, et al. 2025). Four weeks of COS intervention significantly increased intestinal butyrate levels, reduced fasting blood glucose, increased islet area, and alleviated obesity and insulin resistance in db/db mice. These effects were associated with elevated short‐chain fatty acids levels, reshaped microbiota composition, and reduced systemic inflammation (Ji et al. 2022). Accordingly, we hypothesized that COS supplementation could reshape gut microbiota, improve graft survival, and attenuate islet inflammation, thus serving as an adjuvant strategy to promote recovery after ICT.

The study aimed to investigate the relationship between gut dysbiosis and post‐transplant islet function in T2DM recipients, and to clarify the efficacy of COS intervention in the targeted modulation of beneficial gut microbiota. Earlier work has identified marked reductions in *Akkermansia*, *Faecalibacterium*, and *Bacteroides* in the gut microbiota of T2DM patients undergoing ICT, and these three genera effectively improved islet function, leading to the establishment of the MicroAFB Consortium. FMT from patients into antibiotic‐pretreated mice indicated that glycemic recovery after ICT was closely linked to gut microbiota disorders. We further treated HMA‐T2DM mice with COS and the MicroAFB Consortium and evaluated their effects on glycemic control, insulin sensitivity, and intestinal permeability. We then examined the functional targets through which COS improved glucose metabolism under immunosuppression. Finally, functional analyses in HMA‐T2DM‐ICT mice confirmed the beneficial effects of COS on glucose homeostasis and inflammation, supporting its potential as an adjunctive strategy to improve long‐term ICT outcomes.

## 2 Materials and Methods

### 2.1 Materials

The COS mixture used in this study had a degree of polymerization 2–9, a degree of deacetylation > 95%, purity > 95%, and an average molecular weight 2087 Da. It was prepared by the Research and Development Center for Fermentation Separation and Extraction Technology at East China University of Science and Technology (Hou et al. 2022), and its HPLC chromatogram is presented in Figure S1.

### 2.2 Subject Enrollment and Sample Collection

A total of 60 volunteers (31 male and 29 female) were recruited in this study, including 10 patients who underwent ICT and 50 patients with T2DM as the control group. All participants were enrolled from the Department of Endocrinology and the Department of Organ Transplantation at Shanghai Changzheng Hospital and signed the informed consent forms. This study was conducted in accordance with the principles outlined in the Declaration of Helsinki and adhered to the standards stipulated by the Ethics Committee of East China University of Science and Technology (Ethical Approval No.: ECUST‐2022‐104).

In this study, specific inclusion and exclusion criteria were established. T2DM patients were enrolled if they met the American Diabetes Association diagnostic criteria, defined by fasting blood glucose (FBG) ≥ 7.0 mmol/L, glycated hemoglobin A1c (HbA1c) ≥ 6.5%, 2‐h plasma glucose ≥ 11.1 mmol/L following an oral glucose tolerance test (OGTT), and random blood glucose ≥ 11.1 mmol/L in the presence of classic diabetic manifestations (polydipsia, polyuria, and unintentional weight loss). T2DM patients undergoing ICT were included if they were within 3 months of the procedure, aged 18–65 years, receiving postoperative immunosuppressive therapy, insulin‐dependent, and had poor glycemic control (HbA1c > 8.0% or time in range (TIR) < 70%). Patients who met any of the following criteria were excluded: (1) those who received postoperative interventions with gut microbiota‐modulating agents or special dietary regimens (e.g., yogurt consumption); (2) failure to provide study specimens as required or discontinuation of participation during the study; (3) comorbid infection with hepatitis B virus, hepatitis C virus, or other viral hepatitis subtypes, or a diagnosis of hepatobiliary system disorders; (4) female participants who were pregnant, lactating, or in the menstrual phase; (5) participants with a confirmed diagnosis of colitis or other organic gut disorders (Barbe et al. 2026). The final study cohort included 42 T2DM patients and 6 ICT patients (27 male and 21 female), with baseline characteristics detailed in Tables S1 and S2. Fresh fecal specimens were collected into sterile collection tubes immediately after sampling. The obtained samples were snap‐frozen in liquid nitrogen, loaded into dry ice containers for transportation and subsequent analyses (Gemmell et al. 2024).

### 2.3 Animal Experiment

This study strictly adhered to the ARRIVE Guidelines. All procedures involving experimental animals were conducted in accordance with the protocol approved by the Animal Ethics Committee of East China University of Science and Technology (Ethical Approval No.: ECUST‐2022‐061) and complied with the requirements outlined in the Guide for the Care and Use of Laboratory Animals published by the National Research Council. Male C57BL/6 mice (6 weeks old; Jie Sijie Laboratory Animal Co. Ltd., Shanghai, China; Qualification No. 20230004006021) were maintained in a specific pathogen‐free facility under controlled conditions (22°C, 55%–60% relative humidity, 12 h light/12 h dark cycle) in accordance with the 8th edition of the Guide for the Care and Use of Laboratory Animals.

**FIGURE 1:**
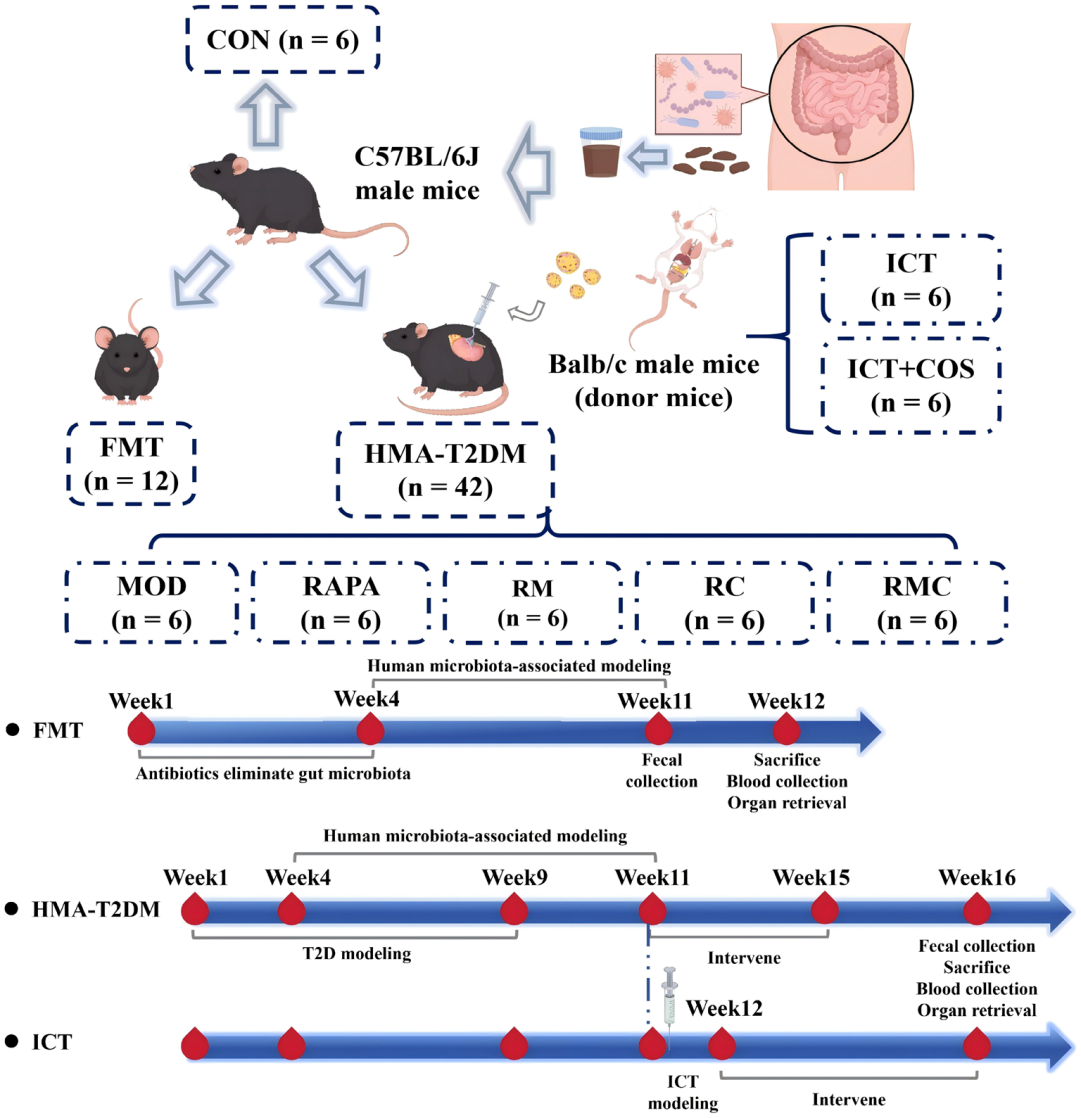
Establishment of the human microbiota‐associated type 2 diabetes mellitus (HMA‐T2DM) mouse model and design of the experimental intervention group.

After a 1‐week acclimatization period, all C57BL/6 mice were randomly allocated to experimental cohorts: the blank control group (CON, *n* = 6), the type 2 diabetes group (T2DM, *n* = 42), and the fecal microbiota‐humanized group (FMT, *n* = 12). The detailed experimental design is illustrated in Figure 1. CON and FMT mice received standard chow diet and sterile water, whereas T2DM mice were fed a 60% high‐fat diet (D12492, Research Diets, USA) and administered streptozotocin (STZ) (Sigma, Missouri, USA) via intraperitoneal injection at 110 mg/kg body weight, dissolved in 0.1 M citrate buffer (pH 4.5; Servicebio, Wuhan, China). On days 3 and 7 post‐injection, random blood glucose was measured. T2DM model establishment was confirmed by blood glucose > 16.7 mmol/L. Male mice were used to establish STZ‐induced T2DM models because they exhibit higher sensitivity to islet cytotoxins than female mice (Racine et al. 2024).

Mice received broad‐spectrum antibiotics in sterile drinking water for 4 weeks. The mixed antibiotic solution consisted of ampicillin, metronidazole, and neomycin sulfate at 1.0 g/L each, together with 0.5 g/L vancomycin (Aladdin, Shanghai, China). The prepared solution was sterilized using 0.22 μm filters and refreshed twice every week (Yuan et al. 2022). After 4 weeks, viable bacterial counts of mouse feces were quantified using serial dilution and spread plating on Columbia blood agar plates. The inoculated plates were incubated at 37°C in a temperature‐controlled incubator (Thermo Fisher Scientific, Waltham, MA, USA) for 48 h, and colony counting was subsequently performed. A 90% decrease in viable bacterial loads verified the successful construction of the antibiotic‐pretreated mouse model (Liang et al. 2020; Zhang, Ji, et al. 2025). Following model establishment, antibiotic‐containing drinking water was replaced with sterile ddH_2_O. HMA‐T2DM and FMT mouse models were then established via intragastric administration of 100 μL fecal bacterial suspension per mouse (OD_600_ ≈1), with each mouse receiving microbiota from one patient (Zhang, Tang, et al. 2025). This gavage protocol was repeated every 2 weeks (3 consecutive days per round), for a total of four rounds.

Mice in the FMT group were randomly divided into 2 subgroups (*n* = 6 each): (1) FMTQ: intragastric gavage with fecal bacterial suspension from T2DM patients; (2) FMTH: intragastric gavage with fecal bacterial suspension from post‐ICT patients. HMA‐T2DM mice were randomly assigned to seven subgroups (*n* = 6 each): (a) MOD group: 0.1 mL vehicle every other day; (b) RAPA group: intragastric gavage with RAPA (5 mg/kg, every other day) (O'Shea et al. 2022); (c) RC group: intragastric gavage with RAPA (5 mg/kg, every other day) and COS (350 mg/kg, daily) (You et al. 2022); (d) RM group: intragastric gavage with MicroAFB Consortium (2 × 10^8^ CFU/0.1 mL) every other day (Lee et al. 2022; Xia et al. 2023); (e) RMC group: intragastric gavage with MicroAFB Consortium (2 × 10^8^ CFU/0.1 mL, every other day) and COS (350 mg/kg, daily); (f) ICT group: intragastric gavage with RAPA (5 mg/kg, every other day) post ICT modeling; (g) ICT + COS group: intragastric gavage with RAPA (5 mg/kg, every other day) and COS (350 mg/kg, daily) post ICT modeling. Cage effects were strictly controlled via cohoused controls and random cage assignment to avoid confounding environmental variations with genotype or treatment effects (Walter et al. 2020).

Single‐intervention groups evaluated the independent effect of each intervention, while combined groups assessed whether the two interventions together produced more favorable regulatory effects than the control and model groups. After intervention, orbital blood was collected after 12 h of fasting for serum preparation. Liver, kidney, and pancreatic tissues were harvested and weighed. Tissue samples were divided into two portions upon collection. One portion was immersed in 4% paraformaldehyde for fixation.

### 2.4 Animal Modeling of Islet Cell Transplantation

Donor male Balb/c mice (6–8 weeks old, Qualification No.: 20230004008956) were euthanized by cervical dislocation and disinfected with 75% ethanol. Following aseptic laparotomy to expose the pancreas and common bile duct, the duct was cannulated and perfused with Collagenase V (2 mg/mL, Sigma, USA) for pancreatic inflation. The tissue was then incubated at 37°C for 10 min, at which point digestion was terminated by HBSS application. Islet cells were filtered and purified by density gradient centrifugation with Histopaque and Hanks' solution (Sigma, Missouri, USA). Islets were collected by manual picking (Yuan et al. 2025). Recipient HMA‐T2DM mice were anesthetized with avertin (250 mg/kg, Sigma, USA). The lateral abdomen was depilated and disinfected. Incisions were made to expose the left kidney. Islet cells were injected under the renal capsule. After hemostasis, the kidney was repositioned and the incision was sutured. Mice were warmed until recovery from anesthesia (Yu et al. 2020).

### 2.5 MicroAFB Consortium Culture and Treatments

*Akkermansia muciniphila* (ATCC BAA‐835), *Faecalibacterium prausnitzii* (DSM 107838), and *Bacteroides fragilis* (ATCC 25285) were purchased from Mingzhou Biotechnology Co. Ltd. (Ningbo, China). They were cultured in anaerobic chambers (Shel Lab, Cornelius, USA) at 37°C for 48–72 h. *Faecalibacterium prausnitzii* and *Akkermansia muciniphila* were cultured in CMC medium, while *Bacteroides fragilis* was cultured in GAM medium. The anaerobic workstation was maintained under constant conditions at 37°C, 75% humidity, and an atmosphere of 80% CO_2_, 10% N_2_, and 10% H_2_. Bacterial stock solutions were centrifuged at 5000 ***g*** for 5 min. The pellets were resuspended in sterile PBS to a concentration of 2 × 10^8^ CFU/0.1 mL. The MicroAFB Consortium was formulated based on the absolute quantitative ratio of the three bacteria in healthy populations: *Akkermansia muciniphila* : *Faecalibacterium prausnitzii* : *Bacteroides fragilis* = 2:4:1 (Canton et al. 2024; Glazunova et al. 2025; Ren et al. 2023; Wu, Shen, et al. 2024). Aliquots of the diluted bacterial suspension were aseptically transferred to sterilized centrifuge tubes, sealed, and administered to mice by oral gavage.

### 2.6 Enzyme‐Linked Immunosorbent Assay (ELISA) Assays

Mouse blood samples were centrifuged at 4°C and 1500 ***g*** for 15 min to obtain serum. Serum cytokine levels were then measured, including INS (Cat. No. ml001983), C‐P (Cat. No. ml063022), GLP‐1 (Cat. No. ml201801), HbA1c (Cat. No. ml401824), monocyte chemoattractant protein‐1 (MCP‐1) (Cat. No. ml037840), and interferon‐γ‐inducible protein 10 (IP‐10) (Cat. No. ml101834). For mouse pancreatic tissue sample preparation, accurately weighed pancreatic tissue was homogenized at 4°C and centrifuged at 1000 ***g*** for 15 min. The supernatant was collected to determine the levels of IRS (Cat. No. ml058259), protein kinase B (AKT) (Cat. No. ml058485), phosphoinositide 3‐kinase (PI3K) (Cat. No. ml001929), forkhead box protein O1 (FOXO1) (Cat. No. ml037860), and glycogen synthase kinase 3β (GSK3β) (Cat. No. ml058082). All aforementioned assay kits were purchased from Shanghai Enzyme Linked Biotechnology Co. Ltd. (Shanghai, China).

### 2.7 Microbiota Analysis

Bacterial genomic DNA was extracted from fecal specimens using the TGuide S96 Magnetic Soil/Fecal DNA Kit (Tiangen Biotech, Beijing, China) and subjected to full‐length 16S rRNA gene amplification with primers 27F and 1492R (27F: ARGTTTTGATYNTGGCTCAG; 1492R: TASGGHTACCTTTTTASGACTT). Purified amplicons were sequenced on the PacBio Sequel II platform (BioMarker Technologies, Beijing, China). CCS reads were generated, demultiplexed, and filtered for chimeras using standard SMRT Link and Lima pipelines. Taxonomic assignment was performed using the Silva 132 database (Bindels et al. 2025). Detailed PCR conditions, reagent specifications, and bioinformatic parameters are provided in Appendix S1.

### 2.8 Fecal Metabolomics Analysis

Fecal metabolites were extracted with methanol‐acetonitrile and analyzed by UHPLC‐Q‐Exactive MS (Shimadzu Corporation, Kyoto, Japan; Thermo Fisher Scientific, Waltham, MA, USA) in positive and negative ion modes. Differential metabolites were screened by OPLS‐DA with criteria of *p* < 0.05 and VIP > 1. Detailed chromatographic conditions, QC procedures, and database matching parameters are provided in Appendix S1 (Feng, Zhong, et al. 2025; Feng, Xiao, et al. 2025).

### 2.9 RNA Extraction and Quantitative Real‐Time PCR

Total RNA was extracted using the TransZol Up Plus RNA Kit and reverse‐transcribed with the TransScript All‐in‐One First‐Strand cDNA Synthesis Mix (Transgen, China). qPCR was performed with AceQ SYBR Green Master Mix using gene‐specific primers (Table S3), with GAPDH as the reference gene (Bachman 2013; Hulshoff et al. 2023).

### 2.10 Statistical Analyses

Data were expressed as the mean ± SD. Two‐group differences were assessed by independent *t*‐tests. Multi‐group comparisons were analyzed by one‐way ANOVA followed by the Tukey's multiple comparisons test. Statistical calculations were conducted using GraphPad Prism 10.5 (GraphPad Software, San Diego, USA), and significance was defined as *p* < 0.05.

## 3 Results

### 3.1 FMT From Patients With Islet Cell Transplantation Induced Glucose Metabolism Dysregulation in Healthy Mice

**FIGURE 2:**
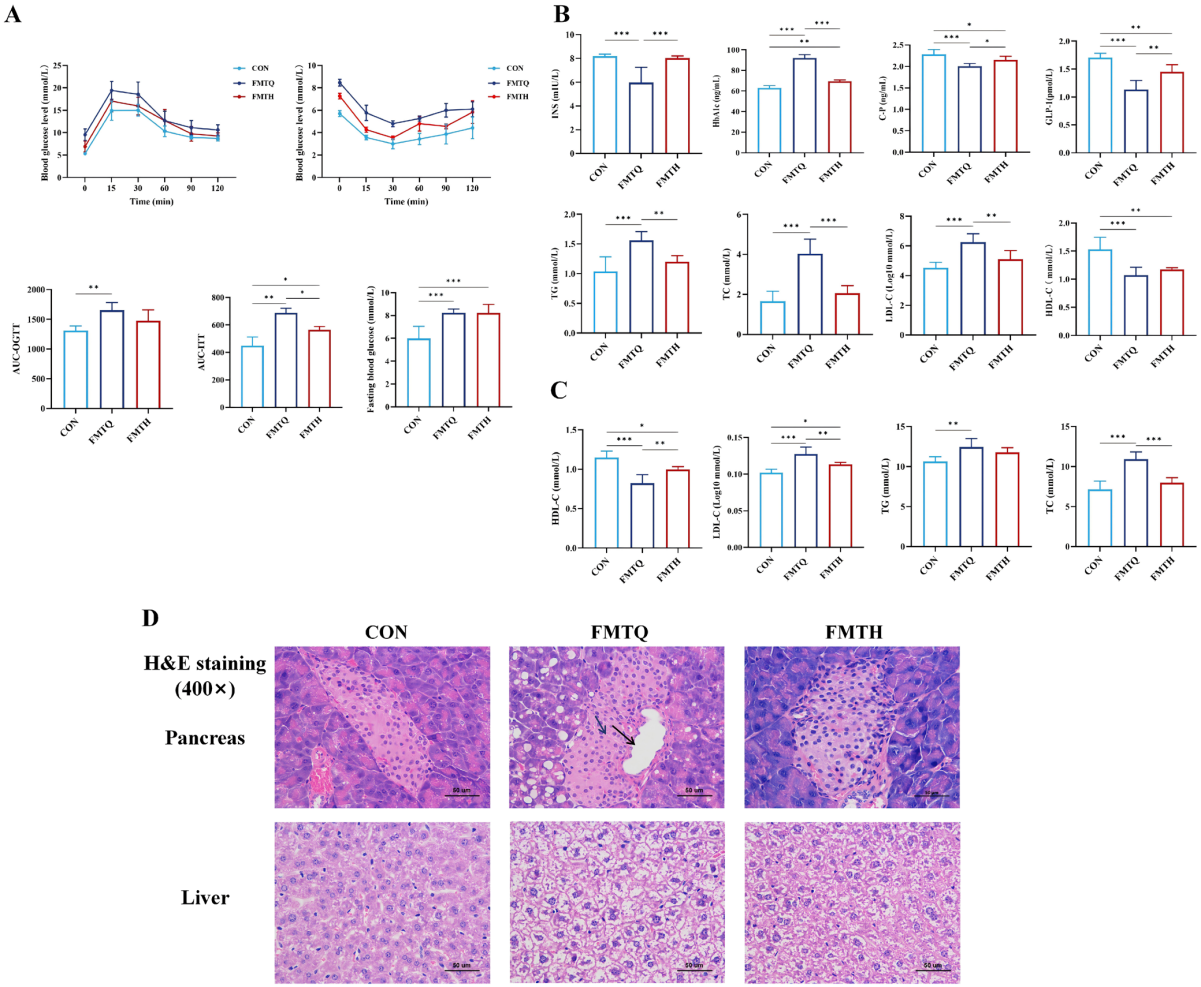
Fecal microbiota transplantation (FMT) from patients' pre‐ and ICT affected blood glucose levels and lipid accumulation in healthy antibiotic‐treated mice. (A) Oral glucose tolerance test (OGTT) and corresponding area under the curve (AUC), insulin tolerance test (ITT) and corresponding AUC, as well as fasting blood glucose levels in fecal microbiota‐humanized mice of each group. (B) Serum glucose and lipid metabolic indices in mice of each group. (C) Hepatic lipid metabolic levels in mice of each group. (D) Hematoxylin and Eosin (H&E) staining of pancreatic and hepatic tissues in mice (scale bar = 50 μm). Statistical comparisons among multiple groups were performed using one‐way ANOVA followed by the Tukey's multiple comparisons test. Values represent the mean ± SD, *n* = 6/group; *p < 0.05, **p < 0.01, and ***p < 0.001.

To investigate whether gut microbiota shifts pre‐ and post‐ICT drive postoperative glycemic, inflammatory, and glycolipid metabolic phenotypic alterations, antibiotic‐treated mice were transplanted with fecal microbiota collected from patients at the two time points. As shown in Figure 2A, fasting glycemic values were substantially elevated in both FMT groups compared with the healthy control group (*p* < 0.001). OGTT demonstrated that blood glucose concentrations were markedly elevated in FMT mice at all time points relative to controls. The area under the curve (AUC) was significantly higher in the FMTQ group than in the CON group (*p* < 0.01) and showed a decreasing trend in the FMTH group compared with the FMTQ group. Insulin tolerance test (ITT) results disclosed compromised insulin sensitivity in FMT mice, with elevated glycemic levels compared to the CON group (*p* < 0.05). The AUC was also significantly diminished in the FMTH group compared with FMTQ (*p* < 0.05). This finding indicated that changes in the gut microbiota after ICT were closely linked to glycemic regulation at the mechanistic level.

To determine whether gut microecological disturbances affect glucose homeostasis and islet function, serum glucose and lipid metabolism parameters were analyzed in FMT mice. As illustrated in Figure 2B, the FMTQ group exhibited significantly attenuated insulin, C‐peptide, GLP‐1, and HDL‐C levels (*p* < 0.001), along with elevated HbA1c, TC, TG and LDL‐C levels (*p* < 0.01) compared with the CON group. Notably, these parameters were partially restored in the FMTH cohort compared with FMTQ, consistent with the postoperative glycemic trajectory. As shown in Figure 2C, hepatic TC, TG and LDL‐C levels were significantly increased (*p* < 0.01) and HDL‐C decreased (*p* < 0.001) in the FMTQ group than in the CON group. Compared with FMTQ, the FMTH group displayed significantly lower LDL‐C (*p* < 0.01) and TC (*p* < 0.001) and higher HDL‐C (*p* < 0.01). These findings demonstrated that FMT effectively reconfigured glucolipid metabolic profiles in recipient mice.

H&E staining (Figure 2D) showed that pancreatic tissue in the CON group maintained intact structure and regular cell morphology. The FMTQ group exhibited obvious pathological injury, including vacuolar degeneration, inflammatory infiltration, fibrosis, acinar damage, and islet rupture and atrophy. These lesions were notably alleviated in the FMTH group. The CON group exhibited regularly arranged hepatocytes with intact normal hepatic lobules in liver tissue. The FMTQ group exhibited severe steatosis and structural disorganization, which were markedly ameliorated in the FMTH group. Overall, FMT from T2DM donors induced pancreatic and hepatic injury, whereas FMT from post‐ICT recipients effectively alleviated these pathological changes.

### 3.2 FMT From T2DM Patients Undergoing ICT Impacted Islet Function and Inflammatory Responses in Healthy Mice

**FIGURE 3:**
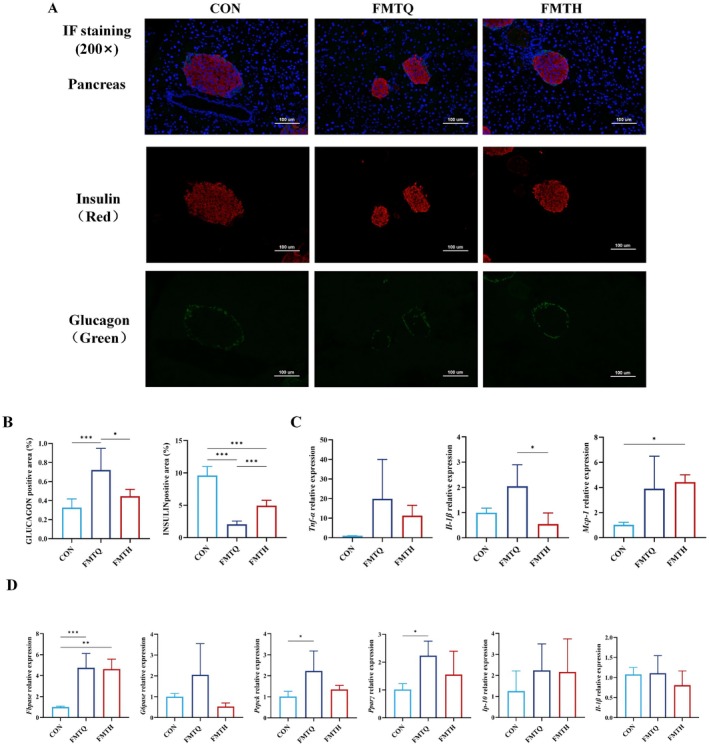
Effects of humanized gut microbiota on glucose metabolic function and islet inflammation‐related mRNA in mice. (A) Immunofluorescence (IF) staining of insulin and glucagon in mouse pancreas (Scale bar, 100 μm). (B) Quantitative area percentage of insulin and glucagon in mice of each group. (C) mRNA expression of pancreatic inflammatory cytokines in mice of each group. (D) Hepatic gluconeogenic and inflammation‐related mRNA expression in mice of each group. Statistical comparisons among multiple groups were performed using one‐way ANOVA followed by the Tukey's multiple comparisons test. Values represent the mean ± SD, *n* = 6/group; *p < 0.05, **p < 0.01, and ***p < 0.001.

To explore the effects of gut microbiota from ICT patients on islet function and inflammation, we analyzed glucose‐ and inflammation‐related genes in mouse pancreas and liver and performed pancreatic immunofluorescence (IF) staining (Figure 3A,B). Relative to the CON group, all FMT groups exhibited significantly diminished insulin secretion (*p* < 0.001). The FMTQ group exhibited a smaller insulin‐positive area, lower β‐cell proportion, and higher α‐cell ratio (*p* < 0.001), indicating islet dysfunction. In contrast, the FMTH group showed restored insulin‐positive area and intensity, and reduced glucagon‐positive signals (*p* < 0.05), suggesting improved islet function. As presented in Figure 3C, the FMTQ cohort showed elevated pancreatic *MCP‐1* and *TNF‐α* expression compared with CON. The *IL‐1β* expression was significantly lower in FMTH than in the FMTQ cohort (*p* < 0.05). These observations verified that gut microbiota from ICT recipients alleviated islet dysfunction and pancreatic inflammation in recipient mice.

To elucidate the mechanism by which alterations in the intestinal microbiota influence glucose homeostasis, hepatic expression levels of glucose metabolism‐related genes were assessed across cohorts (Figure 3D). Hepatic mRNA expression of gluconeogenic enzymes *FBpase* (*p* < 0.001) and *PEPCK* (*p* < 0.05) was significantly elevated in the FMTQ cohort, with a downward trend in the FMTH cohort (*p* > 0.05). Hepatic *PPARγ* maintains glucose homeostasis by regulating lipid metabolism and indirectly modulating insulin sensitivity and glucose signaling (Terra et al. 2023). *PPARγ* expression was significantly upregulated in the FMTQ cohort (*p* < 0.05). Furthermore, hepatic IP‐10 and IL‐1β expression showed a trend toward elevation in the FMTQ cohort and attenuation in the FMTH cohort (*p* > 0.05). These findings indicated that shifts in the intestinal microbiota modulate hyperglycemia, inflammation, and hepatic glucose metabolism in mice.

### 3.3 Fecal Microbiota From ICT Recipients Altered the Gut Microbiota and Barrier Function in Healthy Mice

**FIGURE 4:**
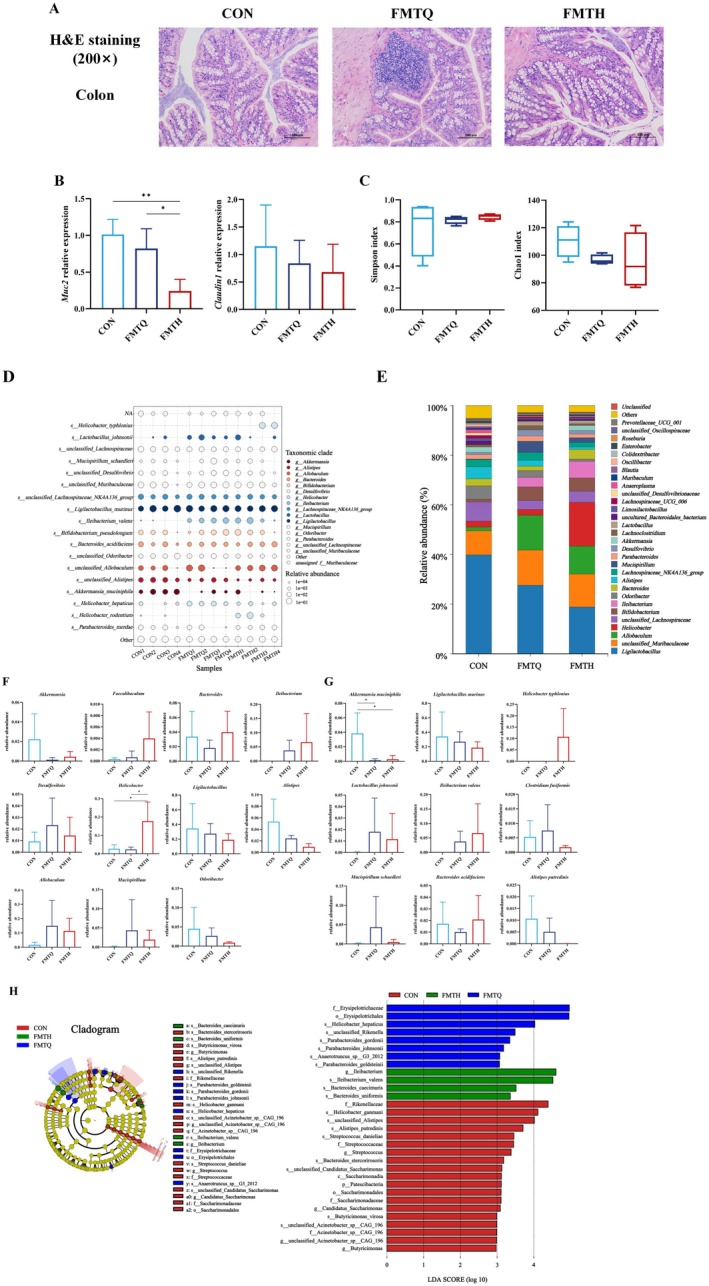
Analysis of colonic barrier integrity and intestinal microbiota in mice across cohorts. (A) H&E staining of the colonic tissue from different treatment cohorts. (Scale bar, 100 μm) (B) Expression of gut barrier‐associated genes in colonic tissue from each cohort. (C) Alpha diversity analysis of gut bacterial communities in mice from each group. (D) Bubble plots for comparison of gut bacterial distribution among mice from different treatment groups. (E) Histograms of gut bacterial distribution at the genus level. (F) Significance analysis of gut bacteria at the genus level. (G) Significance analysis of gut bacteria at the species level. (H) LEfSe analysis of gut bacterial community abundance across treatment cohorts with corresponding LDA score histograms. Statistical comparisons among multiple groups were performed using one‐way ANOVA followed by the Tukey's multiple comparisons test. Values represent the mean ± SD, *n* = 4/group; *p < 0.05, **p < 0.01, and ***p < 0.001.

H&E staining of colonic tissues (Figure 4A) revealed intact epithelium and normal morphology in the CON group. The FMTQ group showed severe pathological damage, including marked inflammatory cell infiltration, disordered glands, and disrupted epithelial integrity. Colon pathology was significantly ameliorated in the FMTH group, though submucosal edema and residual inflammation persisted. As shown in Figure 4B, the mRNA expression of *Muc2* (*p* < 0.01) was markedly reduced in the FMTH group, indicating that the gut barrier was impaired in diabetic model mice. Such impairment could lead to gut bacterial leakage and translocation, thereby inducing islet function impairment. We further examined the effects of pre‐ and post‐ICT fecal microbiota on the intestinal microbiota of recipient mice.

As shown in Figure 4C, α‐diversity reflected by Chao1 and Simpson indices tended to decrease in the FMTQ cohort, and bacterial diversity showed an upward trend following post‐ICT fecal microbiota intervention, with no significant differences observed for either comparison (*p* > 0.05). The Simpson index was higher in the CON group than in the FMTQ and FMTH groups. The Chao1 index was significantly reduced in the FMTQ group but showed a recovery trend in the FMTH group. These results indicated that pre‐ICT FMT decreased gut microbial diversity, whereas post‐ICT transplantation led to a slight recovery. PCA and PCoA analyses showed no significant β‐diversity differences among groups (Figure S2A), while PLS‐DA demonstrated distinct clustering between the CON and intervention cohorts, with FMTH positioned closer to CON. These findings confirmed that ICT gut microbiota intervention optimized microbial structure, which was closely associated with blood glucose and islet function.

Species distribution histograms were used to illustrate the relative abundance of species across groups and to compare intergroup differences (Figure 4D,E). At the genus level, the microbial community structure changed significantly before and after FMT. Mice subjected to post‐ICT fecal microbiota intervention exhibited a trend toward increased relative abundances of *Akkermansia*, *Faecalibaculum*, *Bacteroides* and *Ileibacterium* relative to the FMTQ cohort (*p* > 0.05), whereas *Helicobacter* abundance was significantly increased (*p* < 0.05). After FMT, the relative abundances of *Desulfovibrio*, *Allobaculum*, and *Mucispirillum* were markedly elevated (*p* < 0.05), and the trend of species distribution in the FMTH group was more consistent with that of the CON compared with the FMTQ group (Figure 4F,G).

Linear discriminant analysis effect size (LEfSe) was employed to identify significantly differential bacterial biomarkers between cohorts (Figure 4H). The FMTQ group was predominantly enriched in *Erysipelotrichaceae* (associated with metabolic diseases) (Wu, Lou, et al. 2024), *Helicobacter hepaticus* (associated with liver diseases) (Jin et al. 2025), unclassified *Rikenella* (associated with intestinal inflammation) (Fu et al. 2026), *Parabacteroides gordonii* (anti‐inflammatory attributes) (Abais‐Battad et al. 2021), *Parabacteroides johnsonii* (associated with obesity) (Chen et al. 2025), and *Anaerotruncus* sp. *G3 2012* (associated with gut inflammation) (Zhang et al. 2021). The FMTH group was predominantly enriched in *Ileibacterium* (regulates intestinal immunity) (Wang et al. 2024), *Bacteroides caecimuris* (protects the gut barrier) (Xu et al. 2025), and *Bacteroides uniformis* (regulates intestinal homeostasis) (Yan et al. 2023). Spearman correlation analysis and Mantel tests were conducted to examine associations between intestinal bacteria and disease parameters in the FMTQ and FMTH cohorts, respectively (Figure S2C–E).

### 3.4 Immunosuppressant RAPA Modulated Islet Function and Inflammation in HMA‐T2DM Mice

To verify whether RAPA modulates islet function and inflammatory status in mice recapitulating T2DM pathological features, this study established the HMA‐T2DM mouse model and utilized the functional microbiota MicroAFB Consortium isolated from clinical samples of patients undergoing ICT and identified in our previous research for intervention.

**FIGURE 5:**
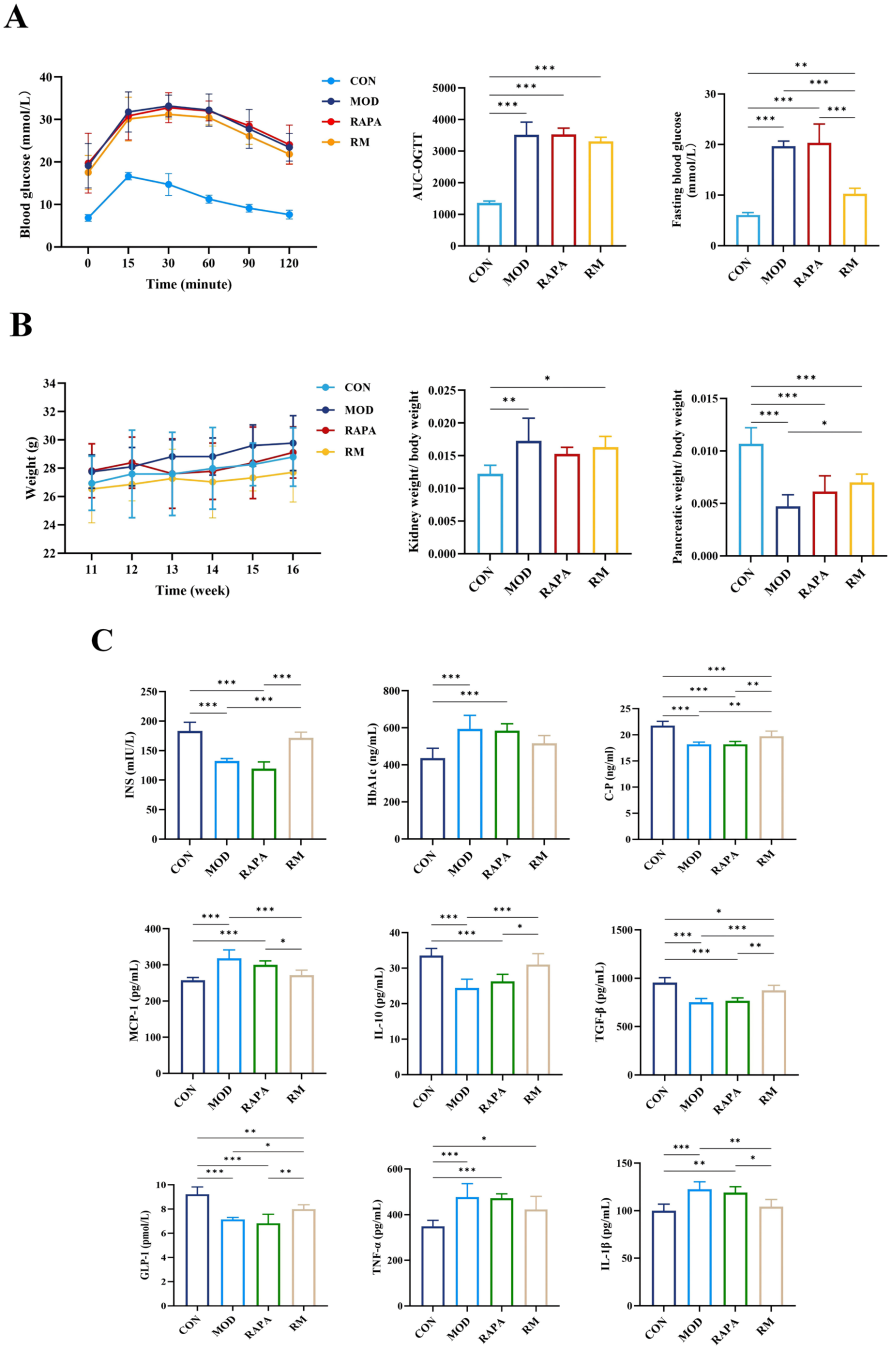
The MicroAFB Consortium ameliorated blood glucose and systemic inflammation in HMA‐T2DM mice affected by RAPA. (A) OGTT, AUC, and fasting blood glucose levels in fecal microbiota‐humanized mice of each group. (B) Body weight and ratios of pancreas and kidney weight to body weight in mice of each group. (C) Levels of islet functional factors and serum inflammatory factors in mice of each group. One‐way ANOVA and Tukey's multiple comparisons test were utilized to compare multiple groups. Data are expressed as the mean ± SD, *n* = 6/group; *p < 0.05, **p < 0.01, and ***p < 0.001.

Glycemic and inflammatory parameters in model mice across treatment cohorts were assessed (Figure 5A). OGTT showed that CON mice maintained stable low blood glucose, whereas MOD and RAPA groups displayed significantly elevated glucose at all time points, indicating impaired glucose metabolism. The RM group exhibited lower glucose levels than the MOD group, suggesting that the MicroAFB Consortium alleviated STZ‐induced glucose dysregulation. RM also significantly reduced fasting blood glucose (*p* < 0.001) and showed a decreasing trend in AUC (*p* > 0.05). As shown in Figure 5B, RAPA intervention reduced body weight. The kidney‐to‐body mass ratio was significantly elevated in the MOD cohort (*p* < 0.01) but attenuated in the RAPA and RM cohorts. The pancreas‐to‐body mass ratio was markedly reduced in the MOD cohort (*p* < 0.001), whereas RM significantly restored this ratio (*p* < 0.05), supporting enhanced pancreatic physiological function. Rapamycin administered at 5 mg/kg every other day represents a widely used preclinical dosing regimen in rodents. This treatment induced adequate immunosuppression alongside acute metabolic stress, which explained the overall reduction in body weight recorded in the present study.

To evaluate glucose homeostasis and chronic low‐grade inflammation, serum biomarkers were measured across four groups (Figure 5C). The MOD group exhibited typical metabolic disorder and inflammatory phenotypes, with significant decreases in INS, C‐P and GLP‐1 (*p* < 0.001) and increases in HbA1c, TNF‐α and IL‐1β (*p* < 0.001). Relative to the MOD group, the RAPA group followed a comparable variation trend with INS showing a tendency to decline (*p* > 0.05). RM intervention significantly improved the declined levels of INS (*p* < 0.001), C‐P (*p* < 0.01) and GLP‐1 (*p* < 0.05), and suppressed the elevation of HbA1c (*p* > 0.05). This treatment tended to lower TNF‐α levels (*p* > 0.05) and significantly suppressed MCP‐1 (*p* < 0.001) and IL‐1β (*p* < 0.01) levels, accompanied by a remarkable increase in the anti‐inflammatory cytokines IL‐10 and TGF‐β (*p* < 0.001). This indicated the MicroAFB Consortium intervention ameliorated the inflammatory status in model mice. It also promoted anti‐inflammatory responses and tissue repair and alleviated glucose metabolic disorders.

### 3.5 MicroAFB Consortium Ameliorated Islet Function and Hepatic Glucose Metabolism in HMA‐T2DM Mice Affected by RAPA

**FIGURE 6:**
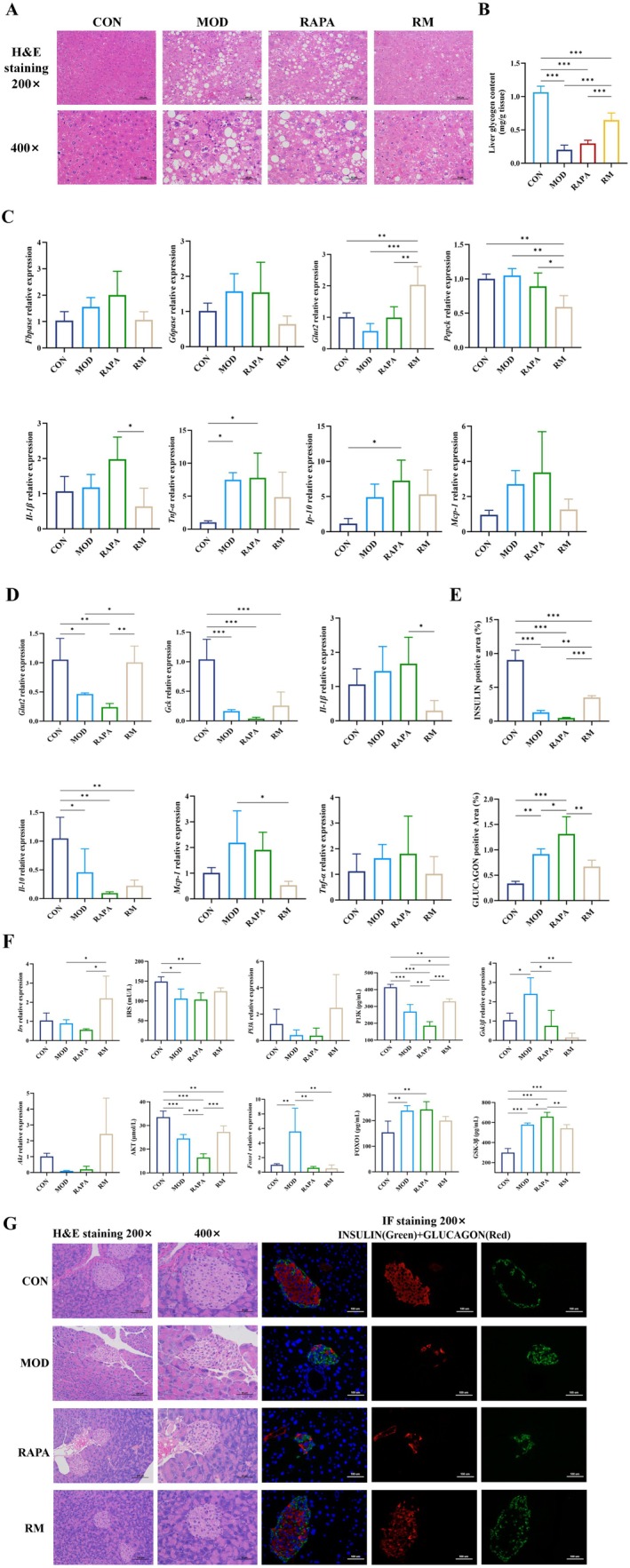
The MicroAFB Consortium ameliorated islet function and hepatic glucose metabolism in HMA‐T2DM mice affected by RAPA. (A) H&E staining of hepatic tissue from different treatment cohorts (Scale bar, 50 μm and 100 μm). (B) Hepatic glycogen content in mice of each cohort. (C) Expression of gluconeogenesis‐related mRNA and inflammatory mediators in hepatic tissue from each cohort. (D) mRNA expression of pancreatic function‐related genes and inflammatory mediators in mice from different treatment cohorts. (E) Quantitative area percentage of insulin and glucagon in mice from each group. (F) mRNA expression and protein level analysis of insulin signaling‐related genes in pancreatic tissue from different treatment cohorts. (G) H&E staining of pancreatic tissues from each cohort (Scale bar, 50 μm and 100 μm) and IF staining of insulin and glucagon (Scale bar, 100 μm). Statistical comparisons among multiple groups were performed using one‐way ANOVA followed by the Tukey's multiple comparisons test. Data are expressed as the mean ± SD, *n* = 6/group for (B), *n* = 4/group for (C–F); *p < 0.05, **p < 0.01, and ***p < 0.001.

Subsequently, islet function and glucose metabolism were examined in each group (Figure 6A,B). H&E staining showed severe hepatocyte steatosis and lipid vacuolation in the MOD group. RAPA treatment exacerbated steatosis and fibrosis, whereas RM markedly reduced lipid deposition and inflammatory infiltration. This indicated that the RAPA aggravated hepatic pathological damage, while the functional microbiota exerted a significant ameliorative effect on hepatic steatosis. Hepatic glycogen content was significantly lower in the MOD and RAPA cohorts than in CON (*p* < 0.001), indicating that hepatic glycogen synthesis and storage were impaired under the MOD condition. RM significantly restored glycogen content relative to both MOD and RAPA (*p* < 0.001).

To explore the molecular mechanisms of RAPA and the MicroAFB Consortium on liver function, hepatic metabolism‐ and inflammation‐related gene expression was analyzed (Figure 6C). The MOD group showed elevated hepatic expression of gluconeogenic genes *FBpase*, *PEPCK*, and *G6pase*. RAPA treatment induced a trend toward elevated *FBpase* expression, which may contribute to aggravated glucose metabolic imbalance (*p* > 0.05). *Glut2* is a key transporter for glucose transmembrane transport, and its deficiency in the liver leads to impaired hepatic glucose uptake and induces postprandial hyperglycemia. RM significantly increased *Glut2* expression (*p* < 0.001) and downregulated *PEPCK* (*p* < 0.01), indicating that the RM group could synergistically ameliorate glucose metabolism disorder by regulating the gluconeogenic pathway. For inflammatory genes, *IL‐1β* and *MCP‐1* levels tended to rise in the MOD and RAPA groups (*p* > 0.05), while *IP‐10* and *TNF‐α* were significantly upregulated (*p* < 0.05), which may facilitate the development of hepatic inflammation. In contrast, RM significantly reduced *IL‐1β* expression (*p* < 0.05) compared with RAPA. These results indicated that RAPA disrupted hepatic metabolic and inflammatory gene expression, while RM effectively ameliorated these abnormalities.

To investigate the molecular mechanisms of different interventions on pancreatic glucose metabolism and inflammation, pancreatic key gene expression was analyzed (Figure 6D). Relative to the CON cohort, *Glut2* (*p* < 0.05) and *Gck* (*p* < 0.001) were significantly decreased in the MOD cohort, and RAPA further aggravated this reduction, indicating impaired β‐cell glucose sensing and insulin secretion. RM treatment significantly upregulated *Glut2* (*p* < 0.05) and restored *Gck* expression compared with the MOD group. The RAPA group showed an upward trend in pancreatic pro‐inflammatory gene expression (*IL‐1β*, *MCP‐1*, *TNF‐α*) (*p* > 0.05) alongside a significant reduction in *IL‐10* expression (*p* < 0.01). By comparison, RM significantly lowered IL‐1β expression (*p* < 0.05) and partially recovered *IL‐10* levels. Overall, RAPA worsened pancreatic glucose metabolic gene downregulation in T2DM mice, whereas functional microbiota partially normalized these abnormalities.

In the HMA‐T2DM mouse model established using the MicroAFB Consortium (Figure 6F), the mRNA expression of insulin receptor substrate (*Irs*) was significantly upregulated (*p* < 0.05), while phosphatidylinositol 3‐kinase (*PI3k*) and protein kinase B (*Akt*) showed upward expression trends without statistical significance (*p* > 0.05). In contrast, the mRNA levels of *Foxo1* and *Gsk3β* were markedly decreased (*p* < 0.01). IRS protein expression was lower in the MOD (*p* < 0.05) and RAPA (*p* < 0.01) cohorts than in the CON cohort, but increased in the RM cohort. Relative to the MOD cohort, RAPA further reduced PI3K (*p* < 0.01) and AKT (*p* < 0.001) proteins while increasing GSK3β (*p* < 0.05). These results indicated that RAPA could exacerbate the impairment of PI3K‐mediated pathway activation and inhibit the gene expression and protein synthesis of AKT. RM treatment improved these changes, activated the insulin signaling pathway, and downregulated FOXO1.

Pancreatic tissues were examined by H&E and IF staining for insulin and glucagon (Figure 6E,G). The MOD group displayed irregular islet morphology, disorganized cells, and obvious tissue damage, which was further aggravated in the RAPA cohort. In contrast, the RM cohort exhibited markedly improved islet structure and cell arrangement. IF staining revealed that the MOD group had fewer insulin‐positive cells and abnormal glucagon distribution. RAPA markedly lowered the proportion of insulin‐positive β‐cells (*p* < 0.001) while elevating the percentage of glucagon‐positive α‐cells (*p* < 0.001). By contrast, RM intervention effectively elevated insulin‐positive cell abundance, normalized glucagon spatial distribution, diminished the relative area of glucagon‐positive cells (*p* < 0.01), and raised that of insulin‐positive cells (*p* < 0.001). In conclusion, RAPA exacerbated pancreatic injury in T2DM mice, while the MicroAFB Consortium protected the pancreas, restored islet α/β‐cell balance, and improved glucose homeostasis.

### 3.6 MicroAFB Consortium Ameliorated Gut Microbiota and Intestinal Barrier in Mice

**FIGURE 7:**
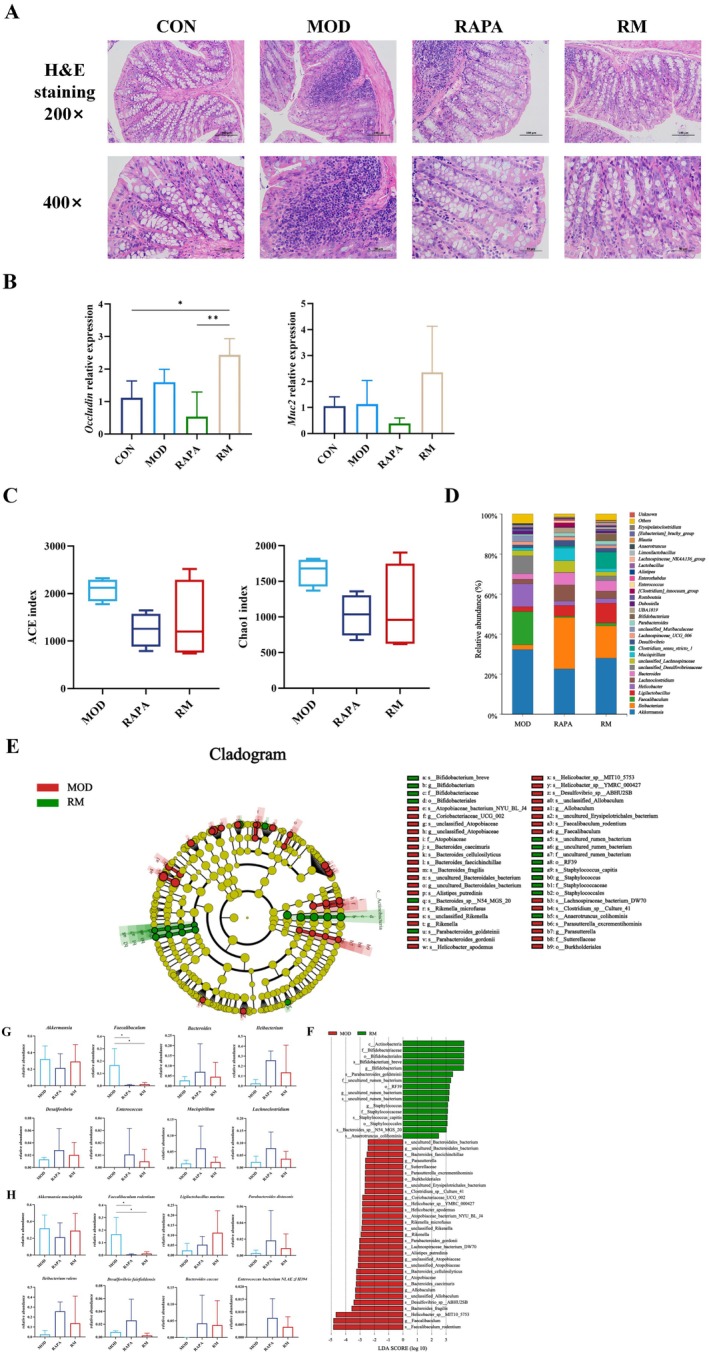
The MicroAFB Consortium improved the intestinal barrier and microbiota in RAPA‐exposed mice. (A) H&E staining of colonic tissue from different treatment cohorts. (Scale bar, 50 μm and 100 μm). (B) Expression of gut barrier‐related genes in colonic tissue from each cohort. (C) Alpha diversity analysis of gut bacterial communities in mice from each group. (D) Histogram of bacterial distribution at the genus level. (E, F) LEfSe analysis of differentially abundant bacterial communities across treatment cohorts. (G) Significance analysis of gut bacteria at the genus level. (H) Significance analysis of gut bacteria at the species level. Statistical comparisons among multiple groups were performed using one‐way ANOVA followed by the Tukey's multiple comparisons test. Values represent the mean ± SD, *n* = 4/group; *p < 0.05, **p < 0.01, and ***p < 0.001.

Colon tissues were analyzed by H&E staining and analysis of gut barrier‐related gene expression (Figure 7A,B). The CON group showed intact colonic epithelium, regular glands, and normal crypts. The MOD group displayed obvious mucosal injury, including disrupted epithelial integrity, disorganized glands, and marked lamina propria inflammatory infiltration. RAPA treatment exacerbated intestinal mucosal injury and inflammatory responses. By contrast, RM intervention effectively preserved epithelial integrity, restored normal glandular morphology, and alleviated inflammatory cell infiltration. *Occludin* was slightly upregulated in the MOD group, significantly downregulated in the RAPA cohort, and markedly restored in the RM cohort (*p* < 0.01). *Muc2* is a mucus secretion‐associated gene that protects the intestinal mucosal barrier (Yao et al. 2021). RAPA treatment led to a decline in *Muc2* expression, while RM intervention induced a trend toward its recovery (*p* > 0.05). In summary, RAPA exacerbated colonic mucosal injury and impaired gut barrier function, while the MicroAFB Consortium effectively improved intestinal permeability and mucosal integrity.

After 4 weeks of intervention, cecal microbiota analysis revealed that the ACE and Chao1 indices exhibited a declining trend in the RAPA cohort (*p* > 0.05), whereas functional microbiota intervention significantly increased gut α‐diversity (Figure 7C). β‐diversity analysis (Figure S2B) revealed distinct differences in gut microbial community structure among the MOD, RAPA, and RM cohorts, with the RM cohort differing substantially from the other two. RAPA reduced microbial richness and disrupted community composition, whereas RM restored microbial diversity and remodeled the gut microbiota structure.

Gut bacterial composition at the genus levels was compared across groups (Figure 7D). Key genera linked to gut barrier function and metabolism, including *Akkermansia* and *Bacteroides*, showed significant intergroup differences. Relative to the MOD cohort, the RAPA cohort showed reduced relative abundances of *Akkermansia* (MOD: RAPA, 32%:23%), *Faecalibaculum* (MOD: RAPA, 17%:0.6%) (*p* < 0.05) and *Helicobacter* (MOD: RAPA, 12%:2.2%). Meanwhile, the relative abundances of *Ileibacterium* (MOD: RAPA, 2.4%:26%), *Ligilactobacillus* (MOD: RAPA, 2.4%:5.4%), *Lachnoclostridium* (MOD: RAPA, 2.2%:8.1%), *Desulfovibrio* (MOD: RAPA, 1.3%:2.9%) and *Mucispirillum* (MOD: RAPA, 1.3%:6.4%) were elevated in the RAPA cohort, with no statistically significant differences observed for these comparisons (*p* > 0.05). The pathogenic bacteria with markedly increased abundances such as *Helicobacter typhlonius* , *Helicobacter rodentium* , and *Desulfovibrio fairfieldensis*, as well as opportunistic pathogenic bacteria, including *Clostridium fusiformis*, *Mucispirillum schaedleri* , and *Enterococcus* bacterium *NLAE zl H394*, exert detrimental effects on the host under conditions of gut barrier impairment and immunodeficiency (Figure S2F) (Chen et al. 2021; Herp et al. 2021). The RM group exhibited decreased relative abundances of *Akkermansia* (MOD: RM, 32%:28%) and *Faecalibaculum* (MOD: RM, 17%:1.4%), and increased relative abundances of *Ileibacterium* (MOD: RM, 2.4%:16%), *Bacteroides* (MOD: RM, 2.7%:5.2%), *Ligilactobacillus* (MOD: RM, 2.4%:9.9%) and *Parabacteroides* (MOD: RM, 0.8%:1.7%). These changes restored dysregulated genera, enriched beneficial taxa, optimized microbial structure, and preserved gut microecological homeostasis. The quantitative results of the three strains in the MicroAFB consortium obtained by qPCR in Figure S2G further verified the observed microbiota variation trends.

LEfSe analysis identified differential gut bacterial biomarkers between the MOD and RM groups (Figure 7E,F). The RM group was enriched in *Actinobacteria*, *Bifidobacterium*, *Staphylococcus*, *Parabacteroides goldsteinii* , *Anaerotruncus colihominis* , whereas the MOD cohort was dominated by *Bacteroides*, *Parasutterella*, *Faecalibaculum*, *Rikenella*, and *Atopobiaceae*. The MicroAFB Consortium reshaped the gut microbiota by increasing beneficial taxa and reducing pathogenic‐associated taxa, improving gut and metabolic function. Genus‐ and species‐level comparisons (Figure 7G,H) showed that RAPA significantly decreased *Akkermansia* and *Faecalibaculum* (*p* < 0.05), while the relative abundances of *Ligilactobacillus*, *Helicobacter*, *Bacteroides*, *Enterococcus*, *Mucispirillum*, and *Ileibacterium* tended to increase without significant intergroup differences (*p* > 0.05). RM treatment markedly improved these RAPA‐induced microbial disturbances.

Therefore, while RAPA was essential for preventing post‐ICT rejection, it severely disrupted gut microbiota homeostasis. RAPA elevated the relative abundances of pro‐inflammatory and pro‐infectious pathogenic bacteria in the murine gut (*Enterococcus* bacterium *NLAE zl H394*, *Desulfovibrio fairfieldensis*, *Helicobacter rodentium* , *Helicobacter typhlonius* , and *Mucispirillum schaedleri* ). RAPA reduced core beneficial genera including *Akkermansia* and *Faecalibacterium*, which were depleted in clinical patients. These changes disturbed host glucose metabolism and impaired the prognosis of transplantation.

### 3.7 COS Ameliorated Glucose Metabolism in HMA‐T2DM Mice Affected by RAPA

**FIGURE 8:**
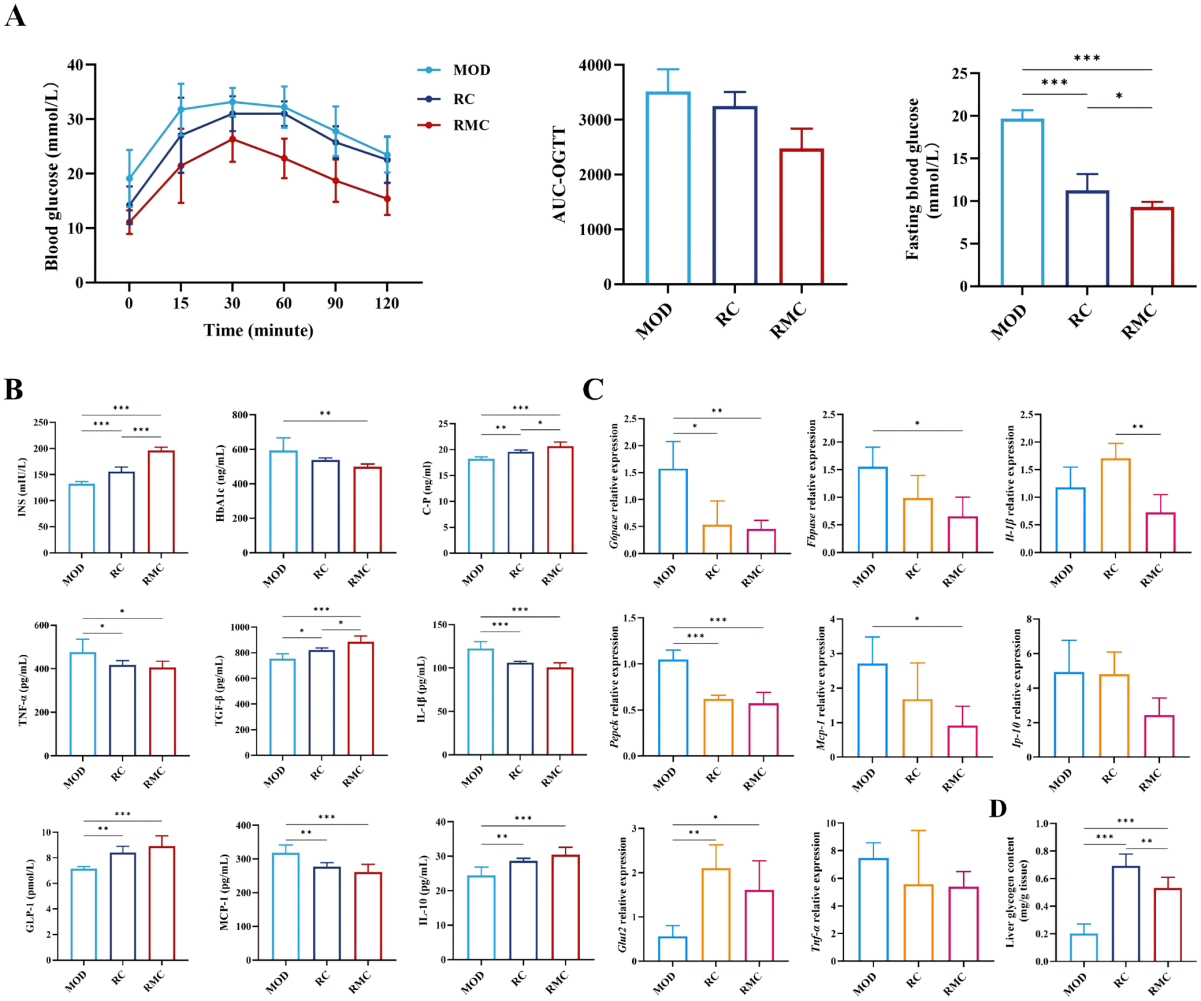
COS ameliorated glucose metabolic disorders and inflammation in RAPA‐exposed HMA‐T2DM mice. (A) OGTT, AUC and fasting blood glucose levels in HMA‐T2DM mice of each group. (B) Serum pancreatic functional factors and inflammatory factor levels in mice of each cohort. (C) Expression of gluconeogenesis‐related mRNA and inflammatory factors in hepatic tissue from each cohort. (D) Hepatic glycogen content in mice of each group. Statistical comparisons among multiple groups were performed using one‐way ANOVA followed by the Tukey's multiple comparisons test. Values represent the mean ± SD, *n* = 6/group; *p < 0.05, **p < 0.01, and ***p < 0.001.

Using an HMA‐T2DM mouse model with the COS and MicroAFB Consortium intervention, we examined the effect of COS on blood glucose under immunosuppression (Figure 8A). Relative to the MOD group, OGTT blood glucose and AUC‐OGTT tended to decrease in the RC and RMC groups (*p* > 0.05), and the RMC group showed a tendency toward better regulation of glucose tolerance and fasting glycemia.

Serum inflammatory and functional factors were measured (Figure 8B). Compared with the MOD group, the COS‐treated RC and RMC groups significantly increased INS (*p* < 0.001), C‐peptide (*p* < 0.01), and GLP‐1 (*p* < 0.01), improving islet β‐cell function and incretin effects. They also reduced MCP‐1 (*p* < 0.01), IL‐1β (*p* < 0.001), and TNF‐α (*p* < 0.05), while increasing IL‐10 (*p* < 0.01) and TGF‐β (*p* < 0.05). These observations indicated that COS combined with the MicroAFB Consortium alleviated HMA‐T2DM by regulating gut hormones, protecting islet function, and suppressing inflammation.

The mRNA expression of inflammatory factor‐ and glucose metabolism‐related genes in hepatic tissue from each group was detected (Figure 8C). Relative to the MOD cohort, the RC cohort significantly downregulated *G6pase* (*p* < 0.05) and *PEPCK* (*p* < 0.001), and upregulated *Glut2* (*p* < 0.01), but proinflammatory genes remained high. *Glut2* expression in the RC cohort was significantly increased (*p* < 0.01). The RMC group showed similar metabolic improvements and further significantly reduced *IL‐1β* (*p* < 0.01) and *MCP‐1* (*p* < 0.05). These findings indicated that RMC inhibited hepatic gluconeogenesis, improved glucose metabolism, and alleviated the inflammatory microenvironment.

Hepatic histopathology (Figure S3B) and glycogen analysis (Figure 8D) demonstrated that RMC alleviated hepatic steatosis, and both RC and RMC cohorts exhibited significantly higher hepatic glycogen levels than the MOD cohort (*p* < 0.001). These changes exerted hepatoprotective effects, while COS effectively ameliorated hepatic glucose metabolism disorders and attenuated inflammation in HMA‐T2DM mice.

### 3.8 COS Ameliorated Glucose Metabolism Through the IRS/PI3K/AKT Islet Pathway

**FIGURE 9:**
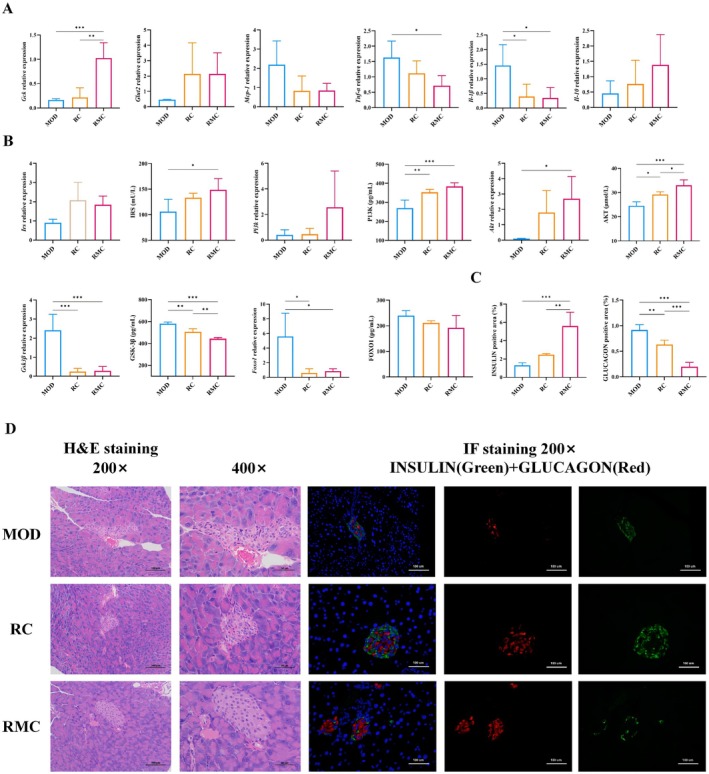
COS ameliorated islet function in HMA‐T2DM mice affected by RAPA. (A) mRNA expression of pancreatic function and inflammatory factors in mice from different treatment groups. (B) mRNA and protein expression of pancreatic insulin signaling‐related genes in mice of each group. (C) Quantitative area percentage of insulin and glucagon in mice of each group. (D) H&E staining (Scale bar, 50 and 100 μm) and IF staining of insulin and glucagon (Scale bar, 100 μm) in the pancreas of mice in each group. Statistical comparisons among multiple groups were performed using one‐way ANOVA followed by the Tukey's multiple comparisons test. Values represent the mean ± SD, *n* = 4/group; *p < 0.05, **p < 0.01, and ***p < 0.001.

We further identified alterations in insulin signaling proteins and downstream glucose metabolism‐related signaling molecules in the pancreas (Figure 9A,B). Compared with the MOD group, both intervention groups upregulated the expression of key glucose metabolism‐related genes *Gck* and *Glut2* and the anti‐inflammatory factor *IL‐10*. These effects were stronger in the RMC group, with more significant *Gck* upregulation (*p* < 0.001) and *TNF‐α* downregulation (*p* < 0.05). Both RC and RMC significantly increased the expression of *Irs*, *PI3k* and *Akt* (*p* < 0.05), and decreased *Gsk3β* (*p* < 0.001) and *Foxo1* (*p* < 0.05). The change trend of insulin signaling pathway protein levels in each group was consistent with that of their mRNA expression. Compared with RC, RMC further elevated AKT (*p* < 0.05) and reduced GSK3β (*p* < 0.01). In conclusion, both interventions activated the IRS/PI3K/AKT/GSK3β/FOXO1 pathway, with RMC showing stronger regulatory effects.

Pancreatic morphology and functional protein expression were evaluated by H&E and dual IF staining for insulin and glucagon (Figure 9C,D). The MOD group showed severely disorganized islets with blurred boundaries and scattered cells. Islet structure was improved in the RC cohort and further restored toward normal in the RMC cohort. These findings indicated that COS protected pancreatic morphology. IF staining revealed weak insulin and glucagon signals in the MOD group, indicating impaired α/β‐cell function. Both RC and RMC treatments markedly enhanced and normalized the fluorescent signals, improving islet cell activity. The RMC cohort significantly increased the insulin‐positive area (*p* < 0.01) relative to the RC cohort, suppressed abnormal glucagon expression, and restored islet α/β‐cell balance. These results demonstrated that COS combined with the MicroAFB Consortium regulated pancreatic glucose metabolism genes and alleviated local inflammation, thereby preserving pancreatic structure and function.

### 3.9 COS Ameliorated Gut Microbiota Dysbiosis in HMA‐T2DM Mice Affected by RAPA

**FIGURE 10:**
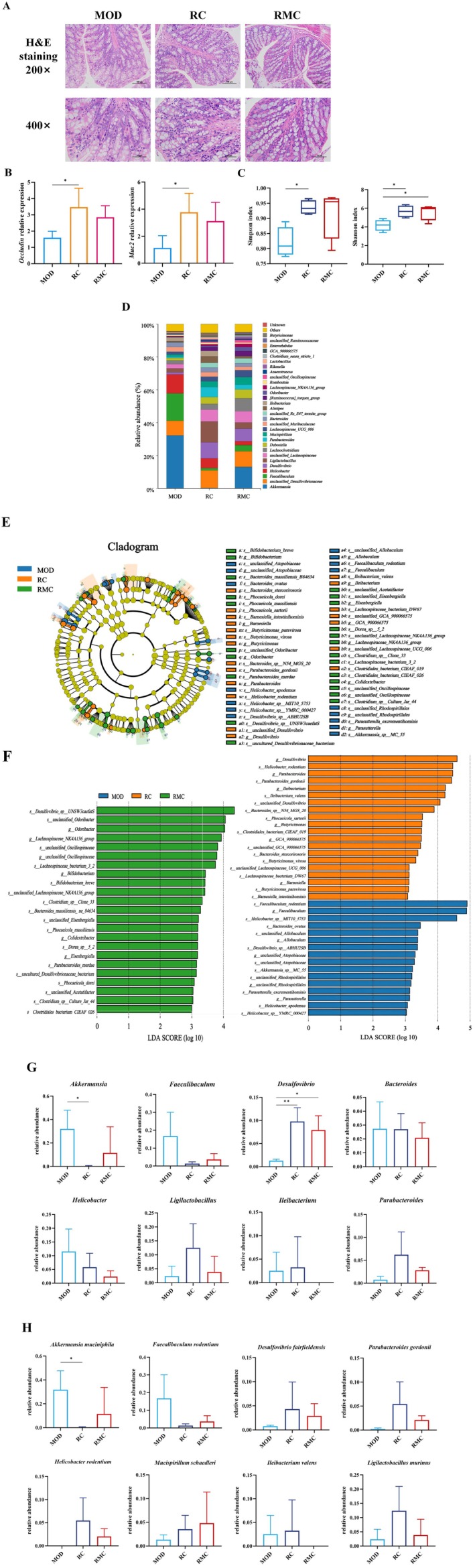
COS ameliorated the intestinal barrier and microbiota in RAPA‐exposed HMA‐T2DM mice. (A) H&E staining of colonic tissue from different treatment cohorts. (Scale bar, 50 μm and 100 μm). (B) expression of barrier‐related genes in colonic tissue from each cohort (C) alpha diversity analysis of the intestinal bacterial community in mice of each cohort (D) bar chart of gut bacterial distribution at the genus level (E, F) LEfSe analysis of differentially abundant bacterial communities across treatment cohorts (*LDA* = 3.0) (G) significance analysis of gut bacteria at the genus level (H) significance analysis of gut bacteria at the species level. Statistical comparisons among multiple groups were performed using one‐way ANOVA followed by the Tukey's multiple comparisons test. Values represent the mean ± SD, *n* = 4/group; *p < 0.05, **p < 0.01, and ***p < 0.001.

Colon tissues were stained with H&E to evaluate gut pathology across groups (Figure 10A). The MOD group exhibited severe colonic mucosal damage with disrupted crypts and massive inflammatory infiltration. The RC group showed partial crypt repair and reduced inflammation, while the RMC group exhibited nearly normal mucosal structure, regular morphology, and minimal infiltration. Relative to the MOD cohort, the mRNA expression of *Occludin* and *Muc2* in colonic tissue from the RC cohort was significantly increased (*p* < 0.05), and the RMC group also showed an increasing trend. Both COS and MicroAFB upregulated colonic tight junction protein expression and ameliorated colonic tissue morphology. They preserved gut barrier function, reduced gut permeability, and attenuated harmful substance translocation.

Analysis of the gut microbiota in HMA‐T2DM mice (Figure 10C) revealed that α‐diversity indices were significantly elevated in the RC cohort, and the Shannon index was increased in the RMC cohort (*p* < 0.05). COS alleviated the reduction in gut microbial α‐diversity triggered by T2DM and RAPA intervention and maintained gut homeostasis. β‐diversity analysis showed distinct separation between the RC/RMC and MOD groups, indicating that the interventions markedly reshaped gut microbial structure (Figure S3C).

To further clarify the specific characteristics of gut microbiota regulation by COS interventions, microbial composition was analyzed at the genus and species levels (Figure 10D–F). Consistent with previous findings, RAPA tended to decrease the relative abundances of *Faecalibaculum* and *Akkermansia*. RMC intervention appeared to elevate their relative abundances. LEfSe analysis revealed that the RC cohort was enriched in *Desulfovibrio*, *Parabacteroides*, *Tannerellaceae*, *Ileibacterium*, *Butyricimonas*, and *Barnesiella intestinihominis* , while the RMC group was enriched in *Lachnospiraceae*, *Bifidobacterium*, *Actinobacteria*, and *Phocaeicola massiliensis*. Compared with the MOD group (Figure 10G,H), both RC and RMC significantly increased *Desulfovibrio* (*p* < 0.05), whereas *Helicobacter* tended to decrease (*p* > 0.05). RMC mitigated the reduction in *Akkermansia* observed in the RC cohort. Both interventions elevated SCFAs‐producing *Parabacteroides* and immune‐regulating *Ligilactobacillus*. At the species level, *Desulfovibrio fairfieldensis*, *Parabacteroides gordonii* , and *Ligilactobacillus murinus* exhibited upward abundance trends in both groups, while *Mucispirillum schaedleri* tended to be further enriched in the RMC group.

Therefore, interventions with COS and MicroAFB effectively reshaped the gut microbial structure. They enriched anti‐inflammatory and SCFAs‐producing bacteria (*Akkermansia*, *Bifidobacterium*, *Butyricimonas*, and *Lachnospiraceae*). They also ameliorated systemic inflammation and blood glucose fluctuations. These findings provided critical insights into the mechanisms whereby intestinal microbiota modulation influences host health.

### 3.10 COS Regulated *Bacteroides*, *Faecalibaculum*, and *Clostridium* to Improve Bile Acid Metabolism in HMA‐T2DM‐ICT Mice

**FIGURE 11:**
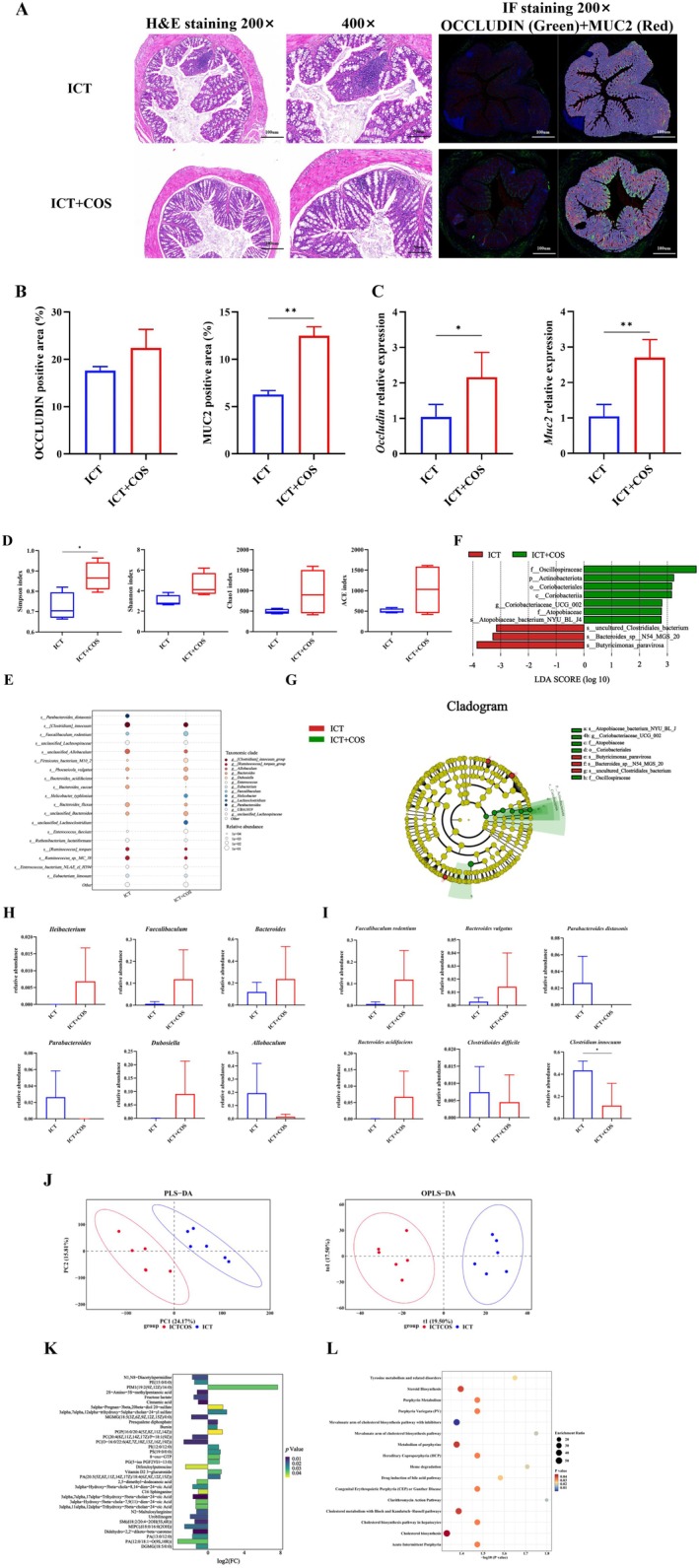
COS ameliorated the intestinal barrier and microbiota in HMA‐T2DM‐ICT mice. (A) H&E staining of mouse colon (scale bars, 50 μm and 100 μm); IF staining of MUC2 and Occludin in the mouse colon (scale bar, 100 μm). (B) Expression of gut barrier‐related genes in mouse colon. (C) Quantitative area percentage of MUC2 and Occludin in mouse colon. (D) Alpha diversity analysis of the gut bacterial community in mice. (E) Bubble plot comparing the gut bacterial distribution between the two groups of mice. (F, G) LEfSe analysis of differences in gut bacterial community abundance among different treatment groups. (H) Significance analysis of gut bacteria at the genus level. (I) Significance analysis of gut bacteria at the species level. (J) OPLS‐DA and PLS‐DA analysis of fecal untargeted metabolome in mice. (K) Bar plot of FC and *p* values for differential metabolites upon COS intervention. (L) KEGG metabolic pathways enriched with differential metabolites in mice upon COS intervention. Pairwise comparisons were performed using the *t*‐test. Values represent the mean ± SD; *p < 0.05, **p < 0.01, and ***p < 0.001.

Colon tissues from ICT and ICT + COS mice were analyzed by H&E and IF staining for MUC2 and Occludin to assess gut pathology (Figure 11A,B). The ICT group exhibited relatively intact yet irregular colonic mucosa, moderately widened intercellular spaces, and mild inflammation. In contrast, the ICT + COS group exhibited well‐preserved colonic structure, regular crypts, and reduced inflammatory infiltration. Occludin showed continuous linear localization, and MUC2 intensity was significantly increased (*p* < 0.01). Consistently, colonic gene expression of *Occludin* (*p* < 0.05) and *Muc2* (*p* < 0.01) expression was upregulated (Figure 11C). These results demonstrated that COS effectively preserved gut barrier structure and function.

To investigate the effects of COS on the intestinal microbiota, 16S rRNA sequencing was performed on mouse cecal contents (Figure 11D). The ICT + COS group showed a significantly higher Simpson index (*p* < 0.05), with increasing trends in the Shannon, ACE, and Chao1 indices. Beta diversity analysis revealed distinct bacterial community structures between the two groups (Figure S3D). Genus‐ and species‐level composition (Figure 11E and Figure S3E) showed that *Bacteroides* (ICT: ICT + COS, 12%:23%), *Faecalibaculum* (ICT: ICT + COS, 6.5%:12%), *Dubosiella* (ICT: ICT + COS, 0.05%:9.0%), *Lachnoclostridium* (ICT: ICT + COS, 0.0013%:6.2%), *Ileibacterium* (ICT: ICT + COS, 0.0%:0.7%) and the *Lachnospiraceae NK4A136 group* (ICT: ICT + COS, 0.0%:1.5%) tended to be enriched in the ICT + COS cohort. In contrast, *Allobaculum* (ICT: ICT + COS, 19%:1.4%), the *Clostridium innocuum group* (ICT: ICT + COS, 44%:12%), the *Ruminococcus torques* *group* (ICT: ICT + COS, 7.0%:0.84%), *Parabacteroides* (ICT: ICT + COS, 2.6%:0.038%) and *Butyricimonas* (ICT: ICT + COS, 2.1%:0.45%) tended to be depleted in this group. Most of these differential genera were involved in SCFA metabolism, gut barrier maintenance, and inflammatory regulation.

LEfSe analysis identified differential gut bacterial biomarkers between the two groups (Figure 11F–I). The ICT group was enriched in *Bacteroides* sp. *N54 MGS 20*, *Butyricimonas paravirosa* and uncultured *Clostridiales bacterium*. The ICT + COS group was enriched in *Oscillospiraceae*, *Actinobacteriota*, *Coriobacteriales*, and *Atopobiaceae*, which were related to gut health, SCFA production, and immune regulation (Zhang et al. 2024). Relative to the ICT group, *Ileibacterium*, *Faecalibaculum*, *Bacteroides* and *Dubosiella* tended to be enriched in the ICT + COS group. *Clostridium innocuum* was significantly depleted (*p* < 0.05), whereas *Parabacteroides distasonis* and *Allobaculum* showed declining trends (*p* > 0.05). COS regulated multiple functional bacteria including *Bacteroides*, *Faecalibaculum*, the *Clostridium innocuum* *group*, and the *Ruminococcus torques* *group*, ameliorated ICT‐induced gut dysbiosis, and restored gut microecological balance.

The gut microbiota and its metabolites critically regulate host metabolism and immune inflammation. COS reshaped the gut bacterial structure and altered metabolite profiles. Untargeted metabolomics based on OPLS‐DA identified 36 differential metabolites (*p* < 0.05), with 12 upregulated and 24 downregulated (Figure 11J,K and Figure S3F). Upregulated metabolites included lipids, signaling molecules, and bile acid sulfate. Downregulated metabolites mainly included bile acid derivatives, phospholipids, amino acids, and microbial metabolites. These results indicated that COS effectively remodeled the gut metabolic profile and improved hepatic bile acid and phospholipid metabolism in post‐ICT mice.

KEGG pathway enrichment showed that COS mainly regulated four key metabolic pathways (Figure 11L): porphyrin and heme metabolism, cholesterol biosynthesis, bile acid and drug metabolism, and tyrosine metabolism. COS exerted concentrated regulatory effects on the porphyrin/heme and cholesterol/bile acid pathways and may modulate lipid and gut metabolism by affecting heme‐dependent cytochrome P450 activity and cholesterol homeostasis.

### 3.11 COS Ameliorated Glucose Metabolism via IRS/PI3K/AKT Signaling and Suppressed Gluconeogenesis to Restore Islet Function After ICT

**FIGURE 12:**
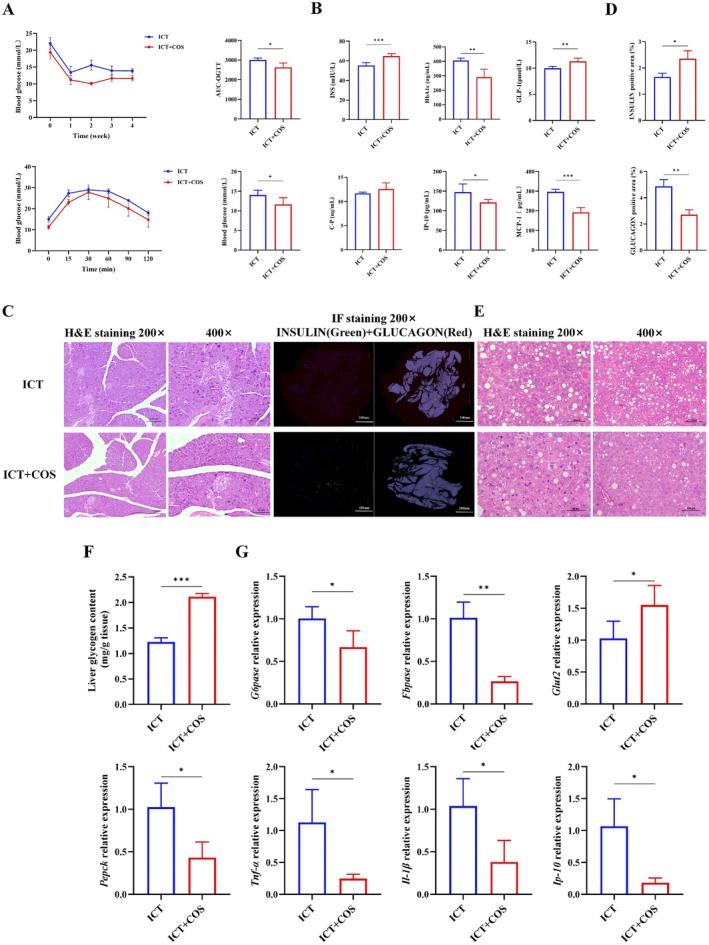
COS ameliorated hepatic and pancreatic functions in mice after ICT. (A) Blood glucose levels of model mice within 4 weeks of COS intervention, OGTT, AUC, and random blood glucose levels at the fourth week. (B) Levels of islet function‐related factors and serum inflammatory factors in mice. (C) H&E staining of pancreatic tissue from the two cohorts (Scale bar, 50 μm and 100 μm), and IF staining of insulin and glucagon (Scale bar, 100 μm). (D) Quantitative area percentage of insulin and glucagon in mice from each group. (E) H&E staining of hepatic tissue (Scale bar, 50 μm and 100 μm). (F) Hepatic glycogen content in mice. (G) mRNA expression of gluconeogenesis‐related genes and inflammatory factors in mouse liver tissues. Pairwise comparisons were performed using the *t*‐test. Values represent the mean ± SD, *n* = 4/group; *p < 0.05, **p < 0.01, and ***p < 0.001.

To investigate the effect of COS on islet functional recovery after ICT, mice in the ICT and ICT + COS groups were monitored for blood glucose, OGTT, and serum factors (Figure 12A,B). Blood glucose dropped in the first week after ICT and fluctuated slightly thereafter. The ICT + COS group showed an earlier and stronger glucose‐lowering effect, with random blood glucose significantly lower than in the ICT cohort (*p* < 0.05), indicating that COS exerted a positive effect on long‐term blood glucose control. In OGTT, the ICT + COS group had a lower glucose peak. The blood glucose level decreased to near the baseline more rapidly within 120 min. The AUC in the ICT + COS cohort was significantly reduced (*p* < 0.05). COS also significantly increased insulin (*p* < 0.001) and GLP‐1 (*p* < 0.01), while reducing HbA1c (*p* < 0.01), MCP‐1 (*p* < 0.001), and IP‐10 (*p* < 0.05). These results demonstrated that COS improved glucose metabolism, enhanced β‐cell function, increased insulin sensitivity, and alleviated chronic inflammation after ICT.

H&E staining of pancreatic tissues (Figure 12C,D) showed that the ICT group maintained relatively intact islet structure but displayed local vacuolation and persistent diabetic injury. The ICT + COS group exhibited significantly improved pancreatic morphology with clear islet boundaries, compact and orderly cell arrangement, and no obvious vacuolar degeneration or inflammatory infiltration. These observations indicated that COS facilitated pancreatic functional recovery. IF staining revealed that the ICT + COS group had significantly stronger and wider insulin fluorescence (*p* < 0.05) and weaker, more restricted glucagon fluorescence (*p* < 0.01) compared with the ICT cohort. These findings demonstrated that COS preserved pancreatic structural integrity and enhanced insulin secretion in β‐cells, supporting its role in improving glucose homeostasis.

To elucidate how COS improve glucose homeostasis, hepatic glycogen content and glucose metabolism‐related gene expression were measured (Figure 12E–G). T2DM is frequently accompanied by hepatic metabolic dysfunction, including enhanced gluconeogenesis, impaired glycogen storage, and lipid accumulation, which aggravate insulin resistance and glycemic instability. These abnormalities further exacerbated insulin resistance and amplified glycemic fluctuations (Uehara et al. 2023), and targeted restoration of hepatic metabolic homeostasis was thus recognized as a key step in halting T2DM progression. Hepatocytes in the ICT group were disorganized, with severe vacuolar degeneration and edema, indicating persistent metabolic dysfunction and hepatic steatosis. The ICT + COS group showed markedly improved hepatocyte morphology, fewer vacuoles, regular cell arrangement, and significantly higher hepatic glycogen content (*p* < 0.001). ICT + COS downregulated hepatic *G6pase* (*p* < 0.05), *FBpase* (*p* < 0.01), and *PEPCK* (*p* < 0.05), and upregulated *Glut2* (*p* < 0.05), suggesting that COS may suppress gluconeogenesis to reduce endogenous glucose production and promote glucose uptake and utilization. The expression of *IL‐1β*, *TNF‐α*, and *IP‐10* was also decreased (*p* < 0.05), alleviating hepatic inflammation.

**FIGURE 13:**
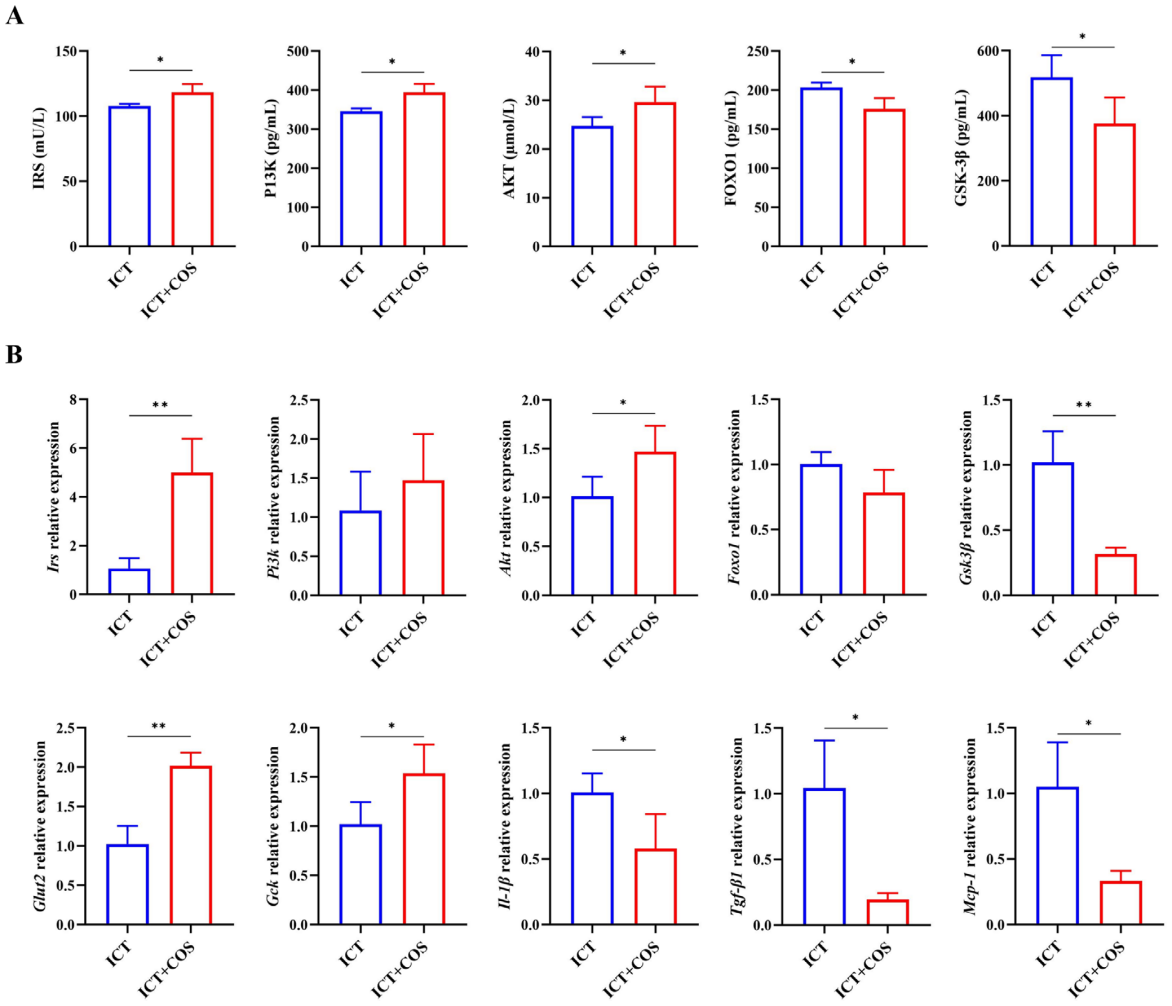
Mechanism of islet function improvement by COS intervention in HMA‐T2DM‐ICT mice. (A) Protein expression related to pancreatic insulin signaling in mice from each group (B) mRNA expression of pancreatic function‐related and inflammatory factors in mice from different treatment groups. Pairwise comparisons were performed using the *t*‐test. Values represent the mean ± SD, *n* = 4/group; *p < 0.05, **p < 0.01, and ***p < 0.001.

To explore the mechanism by which COS regulate islet function, pancreatic protein and inflammatory gene expression were analyzed (Figure 13A,B). ICT + COS treatment significantly upregulated the mRNA expression of *Irs* (*p* < 0.01) and *Akt* (*p* < 0.05), while *PI3k* showed a trend toward upregulation (*p* > 0.05). In addition, *Gsk3β* was significantly downregulated (*p* < 0.01), and *Foxo1* exhibited a declining trend (*p* > 0.05). These molecular alterations may improve insulin signaling and glucose‐stimulated insulin secretion. COS also increased *Glut2* (*p* < 0.01) and *Gck* (*p* < 0.05) to enhance pancreatic glucose sensing. Furthermore, ICT + COS reduced pro‐inflammatory genes *IL‐1β*, *MCP‐1*, and *TGF‐β1* (*p* < 0.05), alleviating pancreatic inflammation. In pancreatic tissues, the protein levels of insulin signaling pathway‐related molecules were consistent with the trend of their corresponding gene expression. Four weeks of COS intervention improved glucose control, stabilized postoperative blood glucose, promoted islet recovery, and reduced pancreatic inflammation in ICT mice. Collectively, these results demonstrated that COS improved post‐operative pancreatic function recovery by targeting the gut microbiota and regulating the IRS/PI3K/AKT and GSK3β/FOXO1 insulin signaling pathways.

## 4 Discussion and Conclusions

Rejection, inflammation and infectious complications after organ transplantation are accompanied by intestinal microecological imbalance, characterized by diminished microbial diversity and increased relative abundance of specific pathogens. Post‐transplant immunosuppression, graft microenvironment and host metabolism jointly shape transplant outcomes. Improving post‐transplant islet performance, reducing drug side effects and extending long‐term graft survival poses a core challenge for diabetes transplant research. Graft dysfunction, immune rejection and glycemic instability are closely linked to intestinal microbial dysbiosis caused by immunosuppressants and antibiotics. A longitudinal analysis of gut bacterial and fungal communities in patients with various solid organ transplants demonstrated that transplantation led to the enrichment of opportunistic pathogens including *Streptococcus* and *Enterococcus*, and the reduction of beneficial bacteria such as *Lachnospira*. A significant positive correlation was identified between *Candida albicans* and *Enterococcus* and *Enterobacter* (Fiedorova et al. 2026). Changes in the gut microbiota strongly link to post‐transplant infection and rejection. Moreover, immunosuppressants, prophylactic antibiotics (e.g., rifaximin), and antifungal agents (e.g., fluconazole) are key clinical factors that drive bacterial‐fungal dysbiosis following transplantation (Swarte et al. 2022). Kidney transplant recipients with acute rejection exhibited markedly lower fecal propionate levels. The correlation between diminished short‐chain fatty acid production and acute rejection links alterations in microbial metabolites to allograft injury (Cho et al. 2025). More severe gut microbiota disruption caused by immunosuppressants predicts higher post‐transplant mortality (Swarte et al. 2020). We thus hypothesize that gut microbiota improvement contributes to postoperative recovery and glycemic control after islet cell transplantation.

Fecal microbiota from T2DM and ICT patients altered glycolipid metabolic profiles in healthy mice. Hepatic and pancreatic tissues showed severe vacuolation and inflammation, with impaired glucose and insulin tolerance, increased intestinal permeability, and downregulated *Muc2* and *Claudin 1*. These results verified that gut microbial dysbiosis after ICT affected islet function and glycemic control, with islet function markers consistent with disease progression. FMT induced distinct functional phenotypic shifts in the intestinal microecology of mice. Preoperative microbiota tended to promote inflammation and metabolic disturbance, whereas postoperative microbiota shifted toward immune regulation and barrier protection. FMT also exhibited significant efficacy in clinical trials. For patients with diarrhea‐predominant irritable bowel syndrome, duodenal FMT relieved abdominal distension. It tended to reduce *Ruminococcus gnavus* and enrich beneficial bacteria represented by *Lawsonibacter*. Meanwhile, microbial hydrogen sulfide synthesis was suppressed after treatment (Aroniadis et al. 2019). These findings confirmed that FMT relieved intestinal symptoms and restored barrier function by reshaping the gut microbiota and their metabolic profiles.

Gut microbiota affects inactivation, prodrug activation, and intestinal absorption of immunosuppressants. The therapeutic response to immune checkpoint inhibitors against tumors correlates with the relative abundance of *Ruminococcaceae* and *Akkermansia muciniphila* , whereas the lipid‐lowering outcomes of statins are modulated by *Bacteroides*. Previous research has demonstrated that prednisolone and mycophenolic acid decrease the relative abundance of *Akkermansia muciniphila* , *Bifidobacterium adolescentis* , and *Eubacterium rectale* , together with multiple SCFAs biosynthesis pathways (Bolte et al. 2025). In humanized gut microbiota T2DM mice treated with RAPA, gut bacterial α‐diversity (ACE, Chao1, Shannon) was significantly decreased. Abundances of core functional bacteria, including *Akkermansia*, *Faecalibacterium*, *Lactobacillus*, *Dubosiella*, and *Limosilactobacillus*, were markedly reduced, whereas opportunistic pathogens such as *Enterococcus*, *Erysipelatoclostridium*, *Desulfovibrio*, and *Mucispirillum* were enriched. These changes were closely associated with impaired glycemic homeostasis and elevated inflammation, indicating that RAPA‐induced gut dysbiosis impairs glucose metabolism and islet function, thus compromising islet transplantation outcomes.

Given the pivotal role of the gut microbiota in drug metabolism and immune modulation, interventions such as probiotics, prebiotics, and FMT enhanced therapeutic benefits and alleviated adverse effects, providing novel adjuvant approaches for clinical practice (Al‐Btoosh et al. 2026). By enriching beneficial bacteria, prebiotics restored the intestinal barrier and immune homeostasis, thus improving post‐transplant outcomes. Prebiotic supplementation after renal transplantation relieved gastrointestinal complications by regulating the gut microbiota (Chan et al. 2023). Inulin supplementation after liver transplantation enriched short‐chain fatty acid‐producing microbes, strengthened intestinal barrier integrity, and mitigated endotoxin‐triggered inflammation (Ma et al. 2021; Singer et al. 2024). COS exerted prominent effects on glycemic control and anti‐inflammatory responses. It activated the CaSR/AMPK signaling pathway to reinforce intestinal barrier integrity, limit intestinal lipid absorption, and attenuate renal inflammatory injury. These protective actions further alleviated insulin resistance and renal lesions in prediabetic rats (Sutthasupha et al. 2022). COS also decreased neutrophil infiltration in colitis and enhanced intestinal mucosal immunity by stimulating SIgA secretion (Wen et al. 2022). Therefore, we administered COS and the MicroAFB Consortium derived from clinical islet transplant recipients to clarify the mechanism by which targeted intestinal microbiota regulation improves host glycemic homeostasis.

The present study revealed that COS significantly ameliorated glucolipid metabolic disorders and inflammation induced by RAPA in mice. In FMT‐ICT mice, COS effectively reduced serum and hepatic glucolipid metabolic parameters and elevated levels of insulin, C‐peptide, and GLP‐1. In HMA‐T2DM and ICT mice, COS also alleviated insufficient insulin secretion, decreased inflammatory factors including IP‐10 and MCP‐1, and improved random blood glucose and glucose intolerance. COS remodeled the intestinal microbial community and increased the abundance of glucose metabolism‐ and immune‐related bacteria such as *Faecalibaculum*, *Bifidobacterium*, *Dubosiella*, *Lachnospiraceae UCG 006*, *Ileibacterium*, and *Lachnospiraceae NK4A136 group*. It further enriched microbial metabolites including secondary bile acids, polyunsaturated fatty acids, and phenolic compounds to enhance host metabolic function.

The IRS/PI3K/AKT pathway is critical for β‐cell survival, insulin secretion, and hepatic gluconeogenesis. Inactivation of this pathway induces insulin resistance and islet dysfunction, whereas excessive activation of the GSK3β/FOXO1 axis suppresses insulin synthesis, elevates hepatic glucose production, and exacerbates hyperglycemia (Camaya et al. 2022). Bioactive components such as natural polysaccharides and dietary fiber activate this pathway via the gut microbiota‐SCFAs axis to improve glucolipid metabolism. Bile acids regulate insulin sensitivity through TGR5/FXR, while SCFAs and other microbial metabolites activate PI3K/AKT signaling to enhance glucose metabolism (Xu et al. 2024). Previous studies demonstrated that fermented dietary fiber activated the hepatic IRS‐1/PI3K/AKT/mTOR pathway, modulated SCFA‐producing bacteria (including *Dubosiella* and *Lactobacillus*), and bile acid metabolism, thereby alleviating hyperglycemia and insulin resistance in mice through the gut‐liver axis and the microbiota‐SCFA‐GPR axis (Mo et al. 2024). Coix seed polysaccharides repaired intestinal barrier integrity, enriched SCFAs‐producing bacteria, and activated the IGF1/PI3K/AKT axis to improve glycemic, insulinemic, and lipid disorders (Xia et al. 2021). Phlorizin relieved free fatty acid‐induced insulin resistance by activating the AMPK/PI3K/AKT pathway (Zhao et al. 2024). *Eucommia ulmoides* leaf‐derived quercetin glycoside enhanced glucose uptake and glycogen synthesis and improved insulin resistance through the IRS‐1/PI3K/AKT/GSK3β pathway (Tang et al. 2023). Diosmetin (Gong et al. 2021) and baicalin (Miao et al. 2024) improved glucose metabolism and insulin resistance in diabetic mice via the IRS/PI3K/AKT axis. Therefore, COS‐mediated modulation of the IRS/PI3K/AKT and GSK3β/FOXO1 pathways contributed to the improvement of pancreatic function in this study.

Collectively, COS remodeled the gut microbiota and mucosal barrier, modulated metabolic and inflammatory pathways, preserved islet β‐cell function and glucolipid homeostasis, and improved islet transplantation outcomes. These findings established a translational route from prebiotic intervention to mechanism‐based therapy for postoperative care and metabolic diseases, supporting the potential of prebiotic agents in intestinal repair and the development of novel adjuvant treatments.

This study revealed that the use of RAPA after ICT led to dysregulation of the gut microbiota and glucose metabolism. By establishing the antibiotic‐treated mice model, fecal microbiota from patients with T2DM and those who had undergone ICT were separately transplanted into mice. Transplantation induced marked hyperglycemia and elevated pancreatic islet inflammation in recipient mice, with pancreatic islet function indices correlating with disease progression. After RAPA intervention in T2DM mice with humanized microbiota, the abundances of core functional bacterial genera including *Akkermansia*, *Faecalibacterium*, *Lactobacillus*, *Dubosiella*, and *Limosilactobacillus* exhibited downward trends in relative abundance. Meanwhile, conditional pathogens such as *Erysipelatoclostridium*, *Desulfovibrio, Enterococcus*, and *Mucispirillum* tended to be enriched among others. Gut microbiota alterations closely correlated with glycemic homeostasis dysregulation and elevated inflammation post‐ICT. This indicated that RAPA‐induced gut microbial dysbiosis was a critical factor for glycemic homeostasis and graft outcomes. On this basis, COS was applied for synergistic intervention in HMA‐T2DM mice, which successfully validated that targeted gut microbiota modulation alleviated RAPA‐triggered intestinal microecological dysbiosis and islet dysfunction after ICT. COS treatment reshaped gut microbiota composition in HMA‐T2DM mice and increased the relative abundance of *Faecalibaculum*, *Bifidobacterium*, *Dubosiella*, and *Lachnospiraceae UCG 006*. Enriched microbiota‐derived metabolites (primarily bile acids and lipids) regulated glucose metabolism in hepato‐pancreatic tissues and the downstream insulin signaling pathway PI3K/AKT/GSK3β/FOXO1. These effects restored pancreatic β‐cell function and ameliorated hepatic gluconeogenesis homeostasis. These findings demonstrated that COS directly improved glucose metabolic function post‐ICT. It also targeted the directed enrichment of the gut microbiota and post‐ICT‐altered core functional bacterial taxa. These dual actions enhanced pancreatic islet function.

However, several limitations remain in the current investigation. The limited sample size of enrolled ICT patients may introduce analytical biases, reduce statistical power and raise the risk of overinterpreting relevant results, restricting the generalizability of our findings. Only one type of immunosuppressant was adopted in this study, the protective effects of COS under other mainstream clinical immunosuppressive regimens such as tacrolimus still need to be validated. The ratio of COS to the microbial consortium requires further optimization. Additionally, efficacy in the HMA mouse model does not fully reflect human physiological responses, and heterogeneity among different transplant populations (e.g., T1DM, T2DM) needs further verification for clinical translation. Further studies should clarify microbial functional alterations through multi‐omics approaches (metatranscriptomics and proteomics), implement well‐designed prospective intervention trials, and evaluate the efficacy and safety of FMT and specific probiotics or prebiotics in transplant recipients. Beyond improving glucose metabolism after ICT, COS intervention suggested a potential strategy to lessen the long‐term requirement for immunosuppressants and antibiotics.

## Author Contributions

**Junfeng Dong:** investigation, data curation. **Duowen He:** methodology. **Hao Yin:** methodology. **Liming Zhao:** conceptualization, methodology, writing – review and editing, funding acquisition, supervision. **Xiaoguo Ji:** writing – review and editing, methodology, data curation. **Shuxin Deng:** investigation, data curation. **Kunlin Chang:** investigation, methodology, formal analysis, writing – original draft, data curation. **Yayu Zhang:** investigation, data curation. **Jiayang Jin:** methodology. **Mengyao Zhao:** methodology.

## Funding

This work was supported by the Shanghai Sailing Program (23YF1409800), the Young Scientists Fund of the National Natural Science Foundation of China (32302102), and the Shanghai Frontiers Science Center of Optogenetic Techniques for Cell Metabolism (Shanghai Municipal Education Commission) and Shanghai Collaborative Innovation Center for Biomanufacturing Technology.

## Conflicts of Interest

The authors declare no conflicts of interest.

## Supporting information


**Table S1:** The clinical data statistics of the enrolled T2DM patients.
**Table S2:** The clinical data statistics of the enrolled ICT patients.
**Table S3:** The qRT‐PCR primer sequences of the mice.
**Figure S1:** The HPLC image of the COS (the horizontal coordinate is min, and the vertical coordinate is LSU).
**Figure S2:** Analysis of gut microflora in mice treated with RAPA and correlation analysis with serum indicators. (A, B) Analysis of the β‐diversity of intestinal bacteria among different groups of mice. (C–E) Spearman correlation analysis and Mantel test were used to analyze the correlations between the differential microorganisms and serum indicators in mice. The comparisons were made as follows: CON group versus FMTQ group, CON group vs. FMTH group, and FMTQ group versus FMTH group. (F) Histogram of gut bacterial distribution at the specie level. (G) Quantitative analysis of mouse fecal bacteria by quantitative real‐time polymerase chain reaction. In the Spearman and Mantel test results, *p* < 0.05 indicates significant differences, while *r*‐values > 0.25 suggest the presence of correlations. One‐way ANOVA and Tukey's multiple comparisons test were utilized to compare multiple groups. Data are expressed as the mean ± SD, *n* = 4/group.
**Figure S3:** Comprehensive impacts of COS intervention on mouse physiology, liver status and gut homeostasis. (A) Body weight, pancreas‐to‐body weight ratio, and liver‐to‐body weight ratio of mice in each group. (B) H&E staining of the liver in mice from different treatment groups (Scale bar, 50 μm and 100 μm). (C, D) Beta diversity analysis of the gut bacterial community in mice from each group. (E) Histogram of gut bacterial distribution at the genus and species level. (F) Volcano plot analysis of fecal metabolites in ICT mice upon COS intervention. One‐way ANOVA and Tukey's multiple comparisons test were utilized to compare multiple groups. Data are expressed as the mean ± SD, *n* = 6/group for (A), *n* = 4/group for (C–E); *p < 0.05, **p < 0.01, and ***p < 0.001.

## Data Availability

The data that support the findings of this study are available from the corresponding author upon reasonable request.
