# Supplementary material for: Microbiota‐Targeted Chitooligosaccharides Intervention Restores Glucose Homeostasis After Islet Cell Transplantation in Rapamycin‐Treated Mice
Source: Food Sci Nutr. 2026 Jul 17;14(7):e72122. doi: 10.1002/fsn3.72122 (PMC13377796; doi:10.1002/fsn3.72122)
Supplement: Supplementary file 1 — Table S1: The clinical data statistics of the enrolled T2DM patients. Table S2: The clinical data statistics of the enrolled ICT patients. Table S3: The qRT‐PCR primer sequences of the mice. Figure S1: The HPLC image of the COS (the horizontal coordinate is min, and the vertical coordinate is LSU). Figure S2: Analysis of gut microflora in mice treated with RAPA and correlation analysis with serum indicators. (A, B) Analysis of the β‐diversity of intestinal bacteria among different groups of mice. (C–E) Spearman correlation analysis and Mantel test were used to analyze the correlations between the differential microorganisms and serum indicators in mice. The comparisons were made as follows: CON group versus FMTQ group, CON group vs. FMTH group, and FMTQ group versus FMTH group. (F) Histogram of gut bacterial distribution at the specie level. (G) Quantitative analysis of mouse fecal bacteria by quantitative real‐time polymerase chain reaction. In the Spearman and Mantel test results, p < 0.05 indicates significant differences, while r‐values > 0.25 suggest the presence of correlations. One‐way ANOVA and Tukey's multiple comparisons test were utilized to compare multiple groups. Data are expressed as the mean ± SD, n = 4/group. Figure S3: Comprehensive impacts of COS intervention on mouse physiology, liver status and gut homeostasis. (A) Body weight, pancreas‐to‐body weight ratio, and liver‐to‐body weight ratio of mice in each group. (B) H&E staining of the liver in mice from different treatment groups (Scale bar, 50 μm and 100 μm). (C, D) Beta diversity analysis of the gut bacterial community in mice from each group. (E) Histogram of gut bacterial distribution at the genus and species level. (F) Volcano plot analysis of fecal metabolites in ICT mice upon COS intervention. One‐way ANOVA and Tukey's multiple comparisons test were utilized to compare multiple groups. Data are expressed as the mean ± SD, n = 6/group for (A), n = 4/group for (C–E); *p < 0.05, **p < 0.01, [file FSN3-14-e72122-s001.docx]

Supplementary Materials for

**Microbiota-Targeted Chitooligosaccharides Intervention Restores Glucose Homeostasis after Islet Cell Transplantation in Rapamycin-Treated Mice**

**1. Materials and Methods**

**1.1 Histological analysis and determination of inflammatory indexes**

Tissue specimens were fixed in 4% paraformaldehyde, dehydrated through graded ethanol series, and embedded in paraffin. Following trimming and sectioning, slides were flattened and baked prior to preparation of 4 μm sections of pancreatic and colonic tissue for H&E staining. Histopathological alterations were examined under an upright optical microscope (Nikon, Tokyo, Japan). Paraffin sections were dewaxed, subjected to antigen retrieval, and blocked with bovine serum albumin. Following overnight incubation with primary antibodies at 4 ℃, sections were incubated with fluorescent secondary antibodies (Insulin, Glucagon, Mucin 2, Occludin) at ambient temperature, followed by DAPI counterstaining. All reagents were obtained from Sevier Biotechnology (Wuhan, China), and images were acquired using a confocal fluorescence microscope (Nikon, Tokyo, Japan).

**1.2 16S rRNA gene sequencing protocol**

The V1-V9 hypervariable regions were amplified using bacterial primers 27F (5'-ARGTTTTGATYNTGGCTCAG-3') and 1492R (5'-TASGGHTACCTTTTTASGACTT-3'). Total PCR amplicons were purified with VAHTS DNA Clean Beads (Vazyme, Nanjing, China) and quantified using the Qubit dsDNA HS Assay Kit with Qubit 3.0 Fluorometer (Invitrogen, Thermo Fisher Scientific, Oregon, USA). Normalized amplicons at equimolar concentrations were pooled and sequenced on the PacBio Sequel II platform using the Sequel II Binding Kit 2.0. Raw subreads were corrected to generate Circular Consensus Sequencing (CCS) reads (SMRT Link, v 8.0). CCS reads from different samples were identified by barcode sequences and chimeras were removed using Lima software (v 1.7.0). Taxonomic annotation was performed against the bacterial 16S Silva database (Release 132, [http://www.arb-silva.de](http://www.arb-silva.de/))

**1.3 Fecal metabolomics analysis**

Precisely weighed 60 mg fecal samples were added to 600 μL methanol-acetonitrile (v/v, containing 4 μg/mL L-2-chlorophenylalanine), ground and ultrasonically extracted for 10 min. After centrifugation at 12000 ×g and 4 ℃ for 10 min, the supernatant was collected and vacuum freeze-dried, then reconstituted with methanol-water (v/v). After re-centrifugation, 200 μL supernatant was collected for subsequent analysis. Quality control (QC) samples were prepared by mixing equal volumes of all sample extracts

Fecal metabolites were separated on a Shimadzu Nexera UHPLC LC-30A system (Shimadzu Corporation, Kyoto, Japan) with chromatographic conditions as previously described Metabolite structures and abundances were identified via a Q-Exactive quadrupole-Orbitrap mass spectrometer (Thermo Fisher Scientific, Waltham, MA, USA) with positive and negative ion scanning modes at an m/z range of 200-1200. Raw data were matched against the HMDB and LIPID MAPS databases. Qualitative analysis and peak area normalization were performed to generate a metabolite data matrix. Intergroup differential metabolites were screened by combining OPLS-DA and Student's t-test with criteria of *p* < 0.05 and VIP >1. Volcano plots (R v 4.1.3; ggplot2 v 3.3.3) were used for visualization of the screening. Subsequent bioinformatic analysis of these differential metabolites was performed on the OmicStudio platform.

**Table S1.** **The clinical data statistics of the enrolled T2DM patients**

| Clinical indicators | T2DM group（n = 42） |
| --- | --- |
|  | Mean ± standard deviation or  median (interquartile range) |
| Gender, n% | 23 (54.7) |
| Age (year) | 56.64 ± 9.77 |
| BMI (kg m-2) | 24.67 ± 2.55 |
| Glycated Hemoglobin (HbA1c, %) | 7.52 ± 0.85 |
| Fasting Blood Glucose (FBG, mmo L-1) | 8.9 (7.7, 9.42) |
| C-Peptide (C-P, nmol L-1) | 0.67 (0.58, 0.96) |
| Insulin (INS, Miu L-1) | 8.07 (5.78, 14.8) |
| Total Cholesterol (TC, mmol L-1) | 3.91 (3.61, 4.97) |
| Triglycerides (TG, mmol L-1) | 1.54 (1.02, 1.92) |
| High-Density Lipoprotein Cholesterol  (HDL, mmol L-1) | 1.38 ± 0.35 |
| Low-Density Lipoprotein Cholesterol  (LDL, mmol L-1) | 2.43 ± 0.89 |
| TNF-α (pg mL-1) | 6.9 (5.4, 8.1) |
| AST/ALT | 1.05 ± 0.31 |
| Uric Acid (UA, μmol L-1) | 307.85 ± 86.83 |

**Table S2. The clinical data statistics of the enrolled ICT patients**

| Clinical indicators | ICT group (n = 6) |
| --- | --- |
|  | Mean ± standard deviation |
| Gender, n% | 4 (66.7) |
| Age (year) | 53.5 ± 11.15 |
| BMI (kg m^-2^) | 23.82 ± 2.45 |
| Bristol Stool Scale Type | 5.00 ± 2.45 |
| Glycated Hemoglobin (HbA1c, %) | 5.92 ± 0.63 |
| Fasting Blood Glucose (FBG, mmo L^-1^) | 6.07 ± 1.85 |
| C-Peptide (C-P, nmol L^-1^) | 0.98 ± 0.43 |
| Insulin (INS, pmol L^-1^) | 66.33 ± 34.23 |
| Total Cholesterol (TC, mmol L^-1^) | 4.12 ± 0.76 |
| Triglycerides (TG, mmol L^-1^) | 1.34 ± 0.52 |
| High-Density Lipoprotein Cholesterol  (HDL, mmol L^-1^) | 1.36 ± 0.52 |
| Low-Density Lipoprotein Cholesterol  (LDL, mmol L^-1^) | 2.04 ± 0.94 |
| Urea (BUN, mmol L^-1^) | 9.43 ± 3.61 |
| Uric Acid (UA, μmol L^-1^) | 409.33 ± 87.43 |
| Creatinine (Cr, μmol L^-1^) | 100.83 ± 24.94 |
| White Blood Cell Count (WBC, ×10^9^μL^-1^) | 6.5 ± 0.51 |
| Neutrophils% | 76.02 ± 3.36 |
| Lymphocytes% | 16.3 ± 4.1 |
| CD3% | 77.63 ± 8.43 |
| CD4% | 48.38 ± 17.2 |
| CD8% | 33.92 ± 14.09 |
| CD4/CD8 | 1.64 ± 0.69 |

**Table S3. The qRT-PCR primer sequences of the mice**

| Gene | Forward (5’-3’) | Reverse (5’-3’) |
| --- | --- | --- |
| *IL-10* | CGGGAAGACAATAACTGCACCC | CGGTTAGCAGTATGTTGTCCAGC |
| *IL-1β* | TGGACCTTCCAGGATGAGGACA | GTTCATCTCGGAGCCTGTAGTG |
| *TNF-α* | GGTGCCTATGTCTCAGCCTCTT | GCCATAGAACTGATGAGAGGGAG |
| *TGF-β* | TGATACGCCTGAGTGGCTGTCT | CACAAGAGCAGTGAGCGCTGAA |
| *G6pase* | AGGTCGTGGCTGGAGTCTTGTC | GTAGCAGGTAGAATCCAAGCGC |
| *Gapdh* | CATCACTGCCACCCAGAAGACTG | ATGCCAGTGAGCTTCCCGTTCAG |
| *FBpase* | AGAGGCAGTGAGCTACAGGAAC | TGACCTTCCTCACGGCTGAGAT |
| *PEPCK* | GGCGATGACATTGCCTGGATGA | TGTCTTCACTGAGGTGCCAGGA |
| *Glut2* | GTTGGAAGAGGAAGTCAGGGCA | ATCACGGAGACCTTCTGCTCAG |
| *IP-10* | ATCATCCCTGCGAGCCTATCCT | GACCTTTTTTGGCTAAACGCTTTC |
| *Akt* | GGACTACTTGCACTCCGAGAAG | CATAGTGGCACCGTCCTTGATC |
| *Gsk3β* | GAGCCACTGATTACACGTCCAG | CCAACTGATCCACACCACTGTC |
| *Claudin1* | CCTGCCCCAGTGGAAGATTT | AAACGCAGGACATCCACAGT |
| *Occludin* | TGGCAAGCGATCATACCCAGAG | CTGCCTGAAGTCATCCACACTC |
| *Muc2* | ATGCCCACCTCCTCAAAGAC | GTAGTTTCCGTTGGAACAGTGAA |
| *Foxo1* | CTACGAGTGGATGGTGAAGAGC | CCAGTTCCTTCATTCTGCACTCG |
| *Irs* | TGTCACCCAGTGGTAGTTGCTC | CTCTCAACAGGAGGTTTGGCATG |
| *PI3k* | CAAACCACCCAAGCCCACTACT | CCATCAGCAGTGTCTCGGAGTT |
| *MCP-1* | GCTACAAGAGGATCACCAGCAG | GTCTGGACCCATTCCTTCTTGG |

**
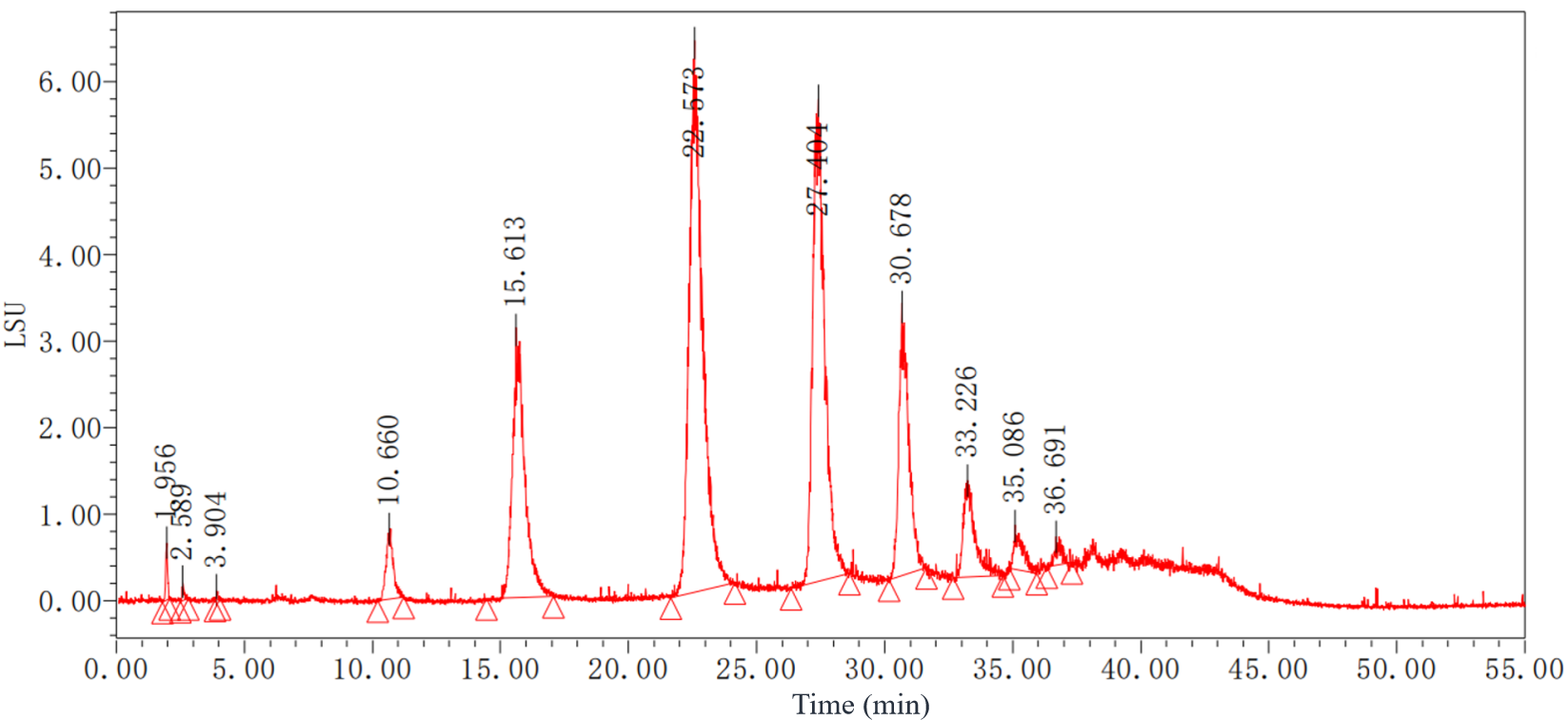
**

**Figure S1. The HPLC image of the COS (the horizontal coordinate is min, and the vertical coordinate is LSU).**


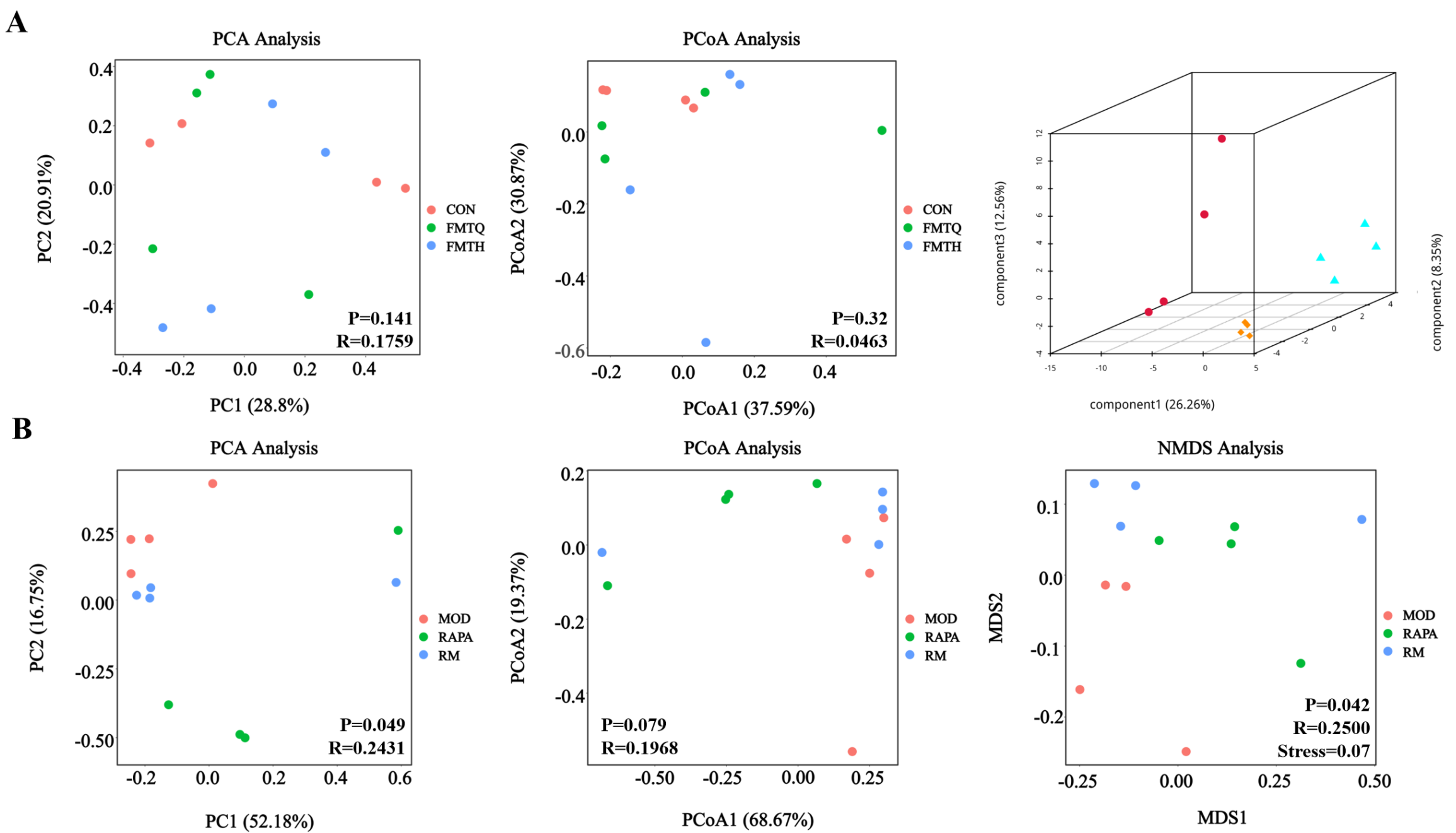

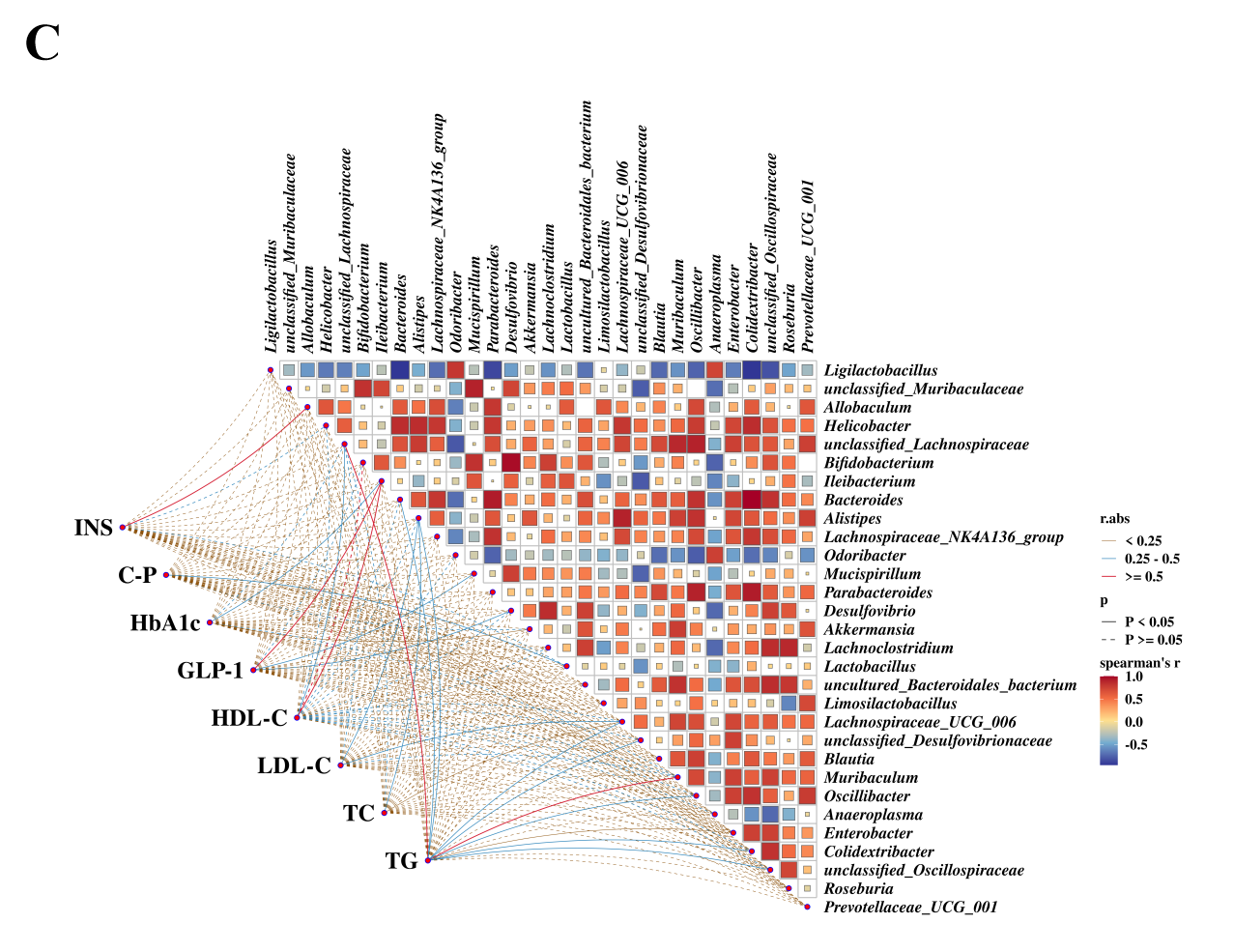

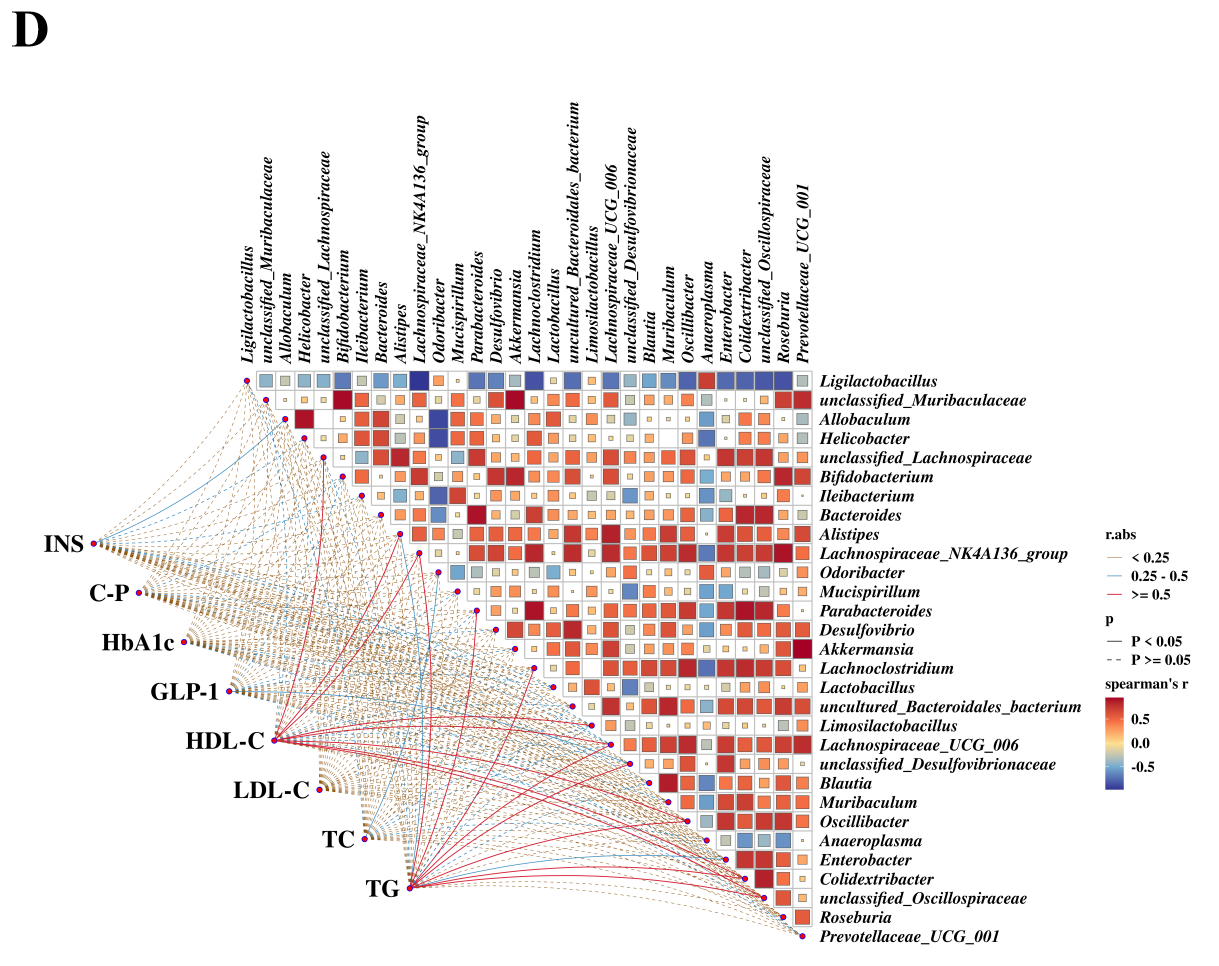

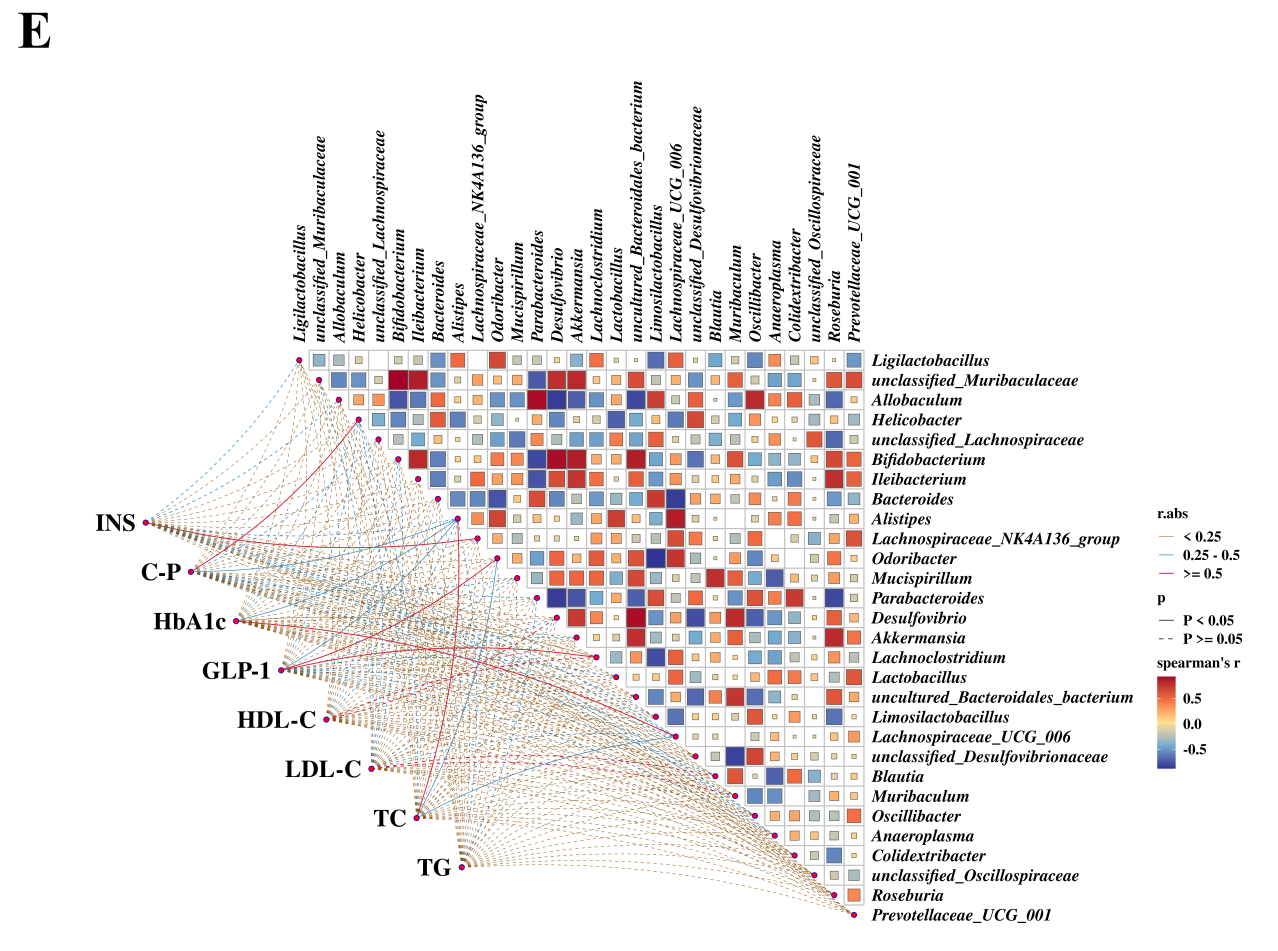


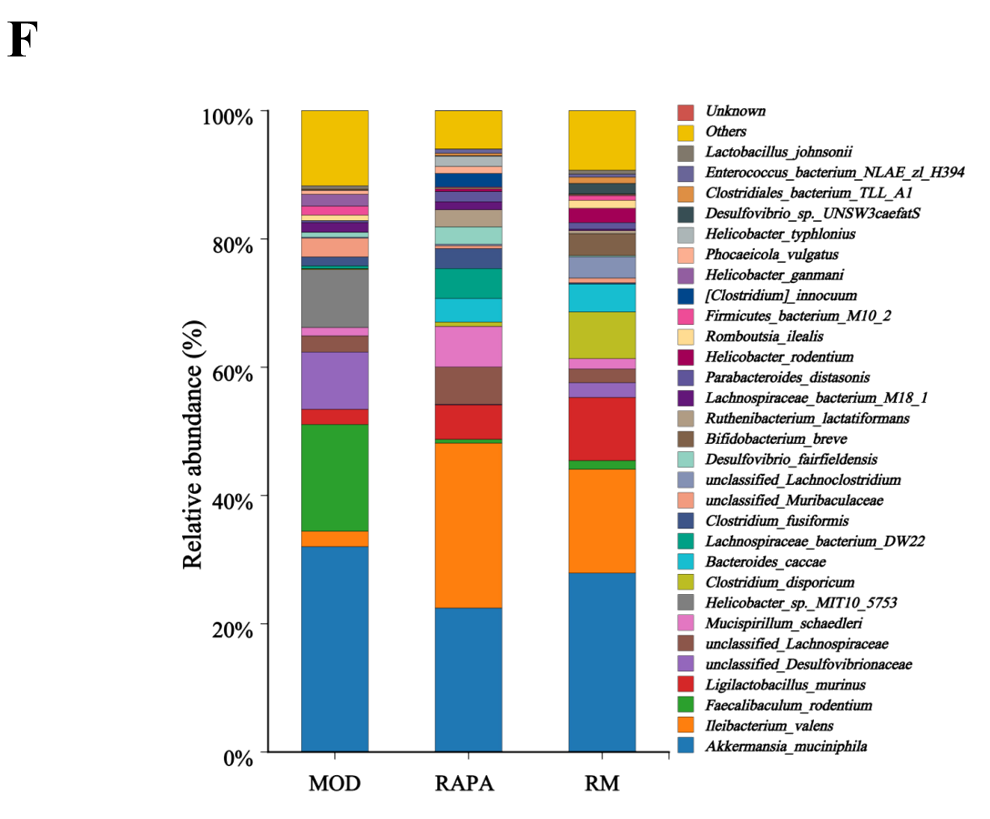


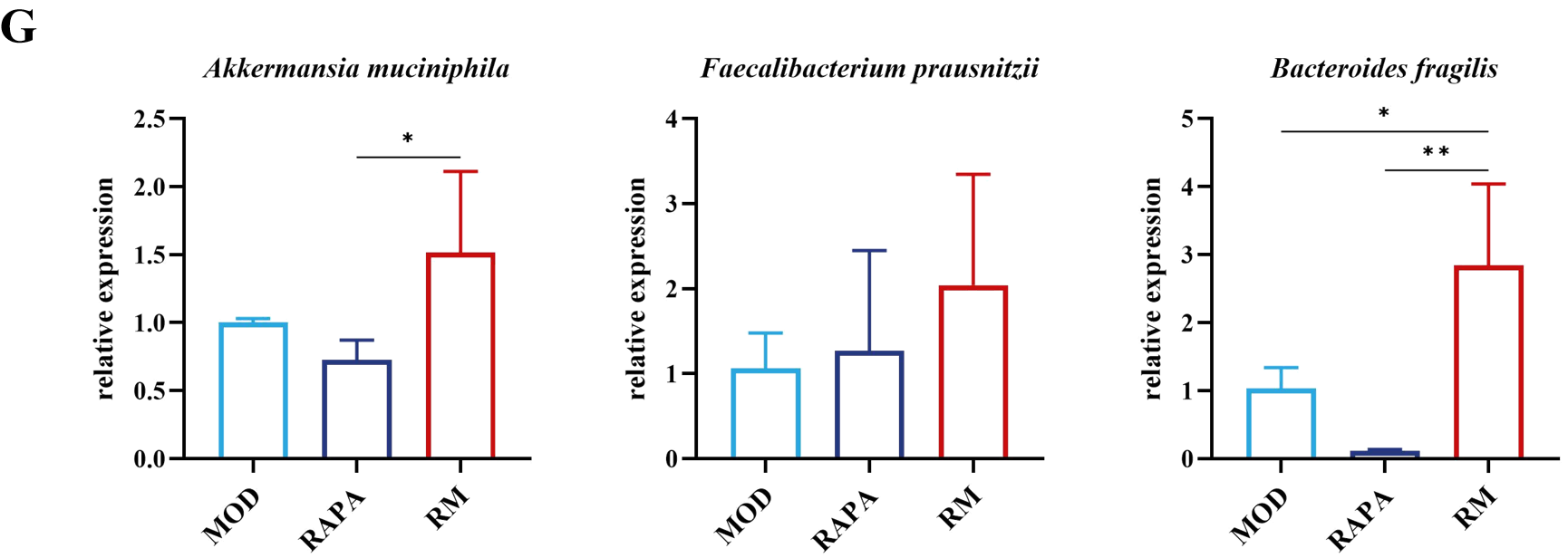


**Figure S2.** **Analysis of gut microflora in mice treated with RAPA and correlation analysis with serum indicators.** (A-B) Analysis of the β-diversity of intestinal bacteria among different groups of mice. (C-E) Spearman correlation analysis and Mantel test were used to analyze the correlations between the differential microorganisms and serum indicators in mice. The comparisons were made as follows: CON group vs. FMTQ group, CON group vs. FMTH group, and FMTQ group vs. FMTH group. (F) Histogram of gut bacterial distribution at the specie level. (G) Quantitative analysis of mouse fecal bacteria by quantitative real-time polymerase chain reaction. In the Spearman and Mantel test results, *p* < 0.05 indicates significant differences, while r-values > 0.25 suggest the presence of correlations. One-way ANOVA and Tukey's multiple comparisons test were utilized to compare multiple groups. Data are expressed as the mean ± SD, n = 4/group.


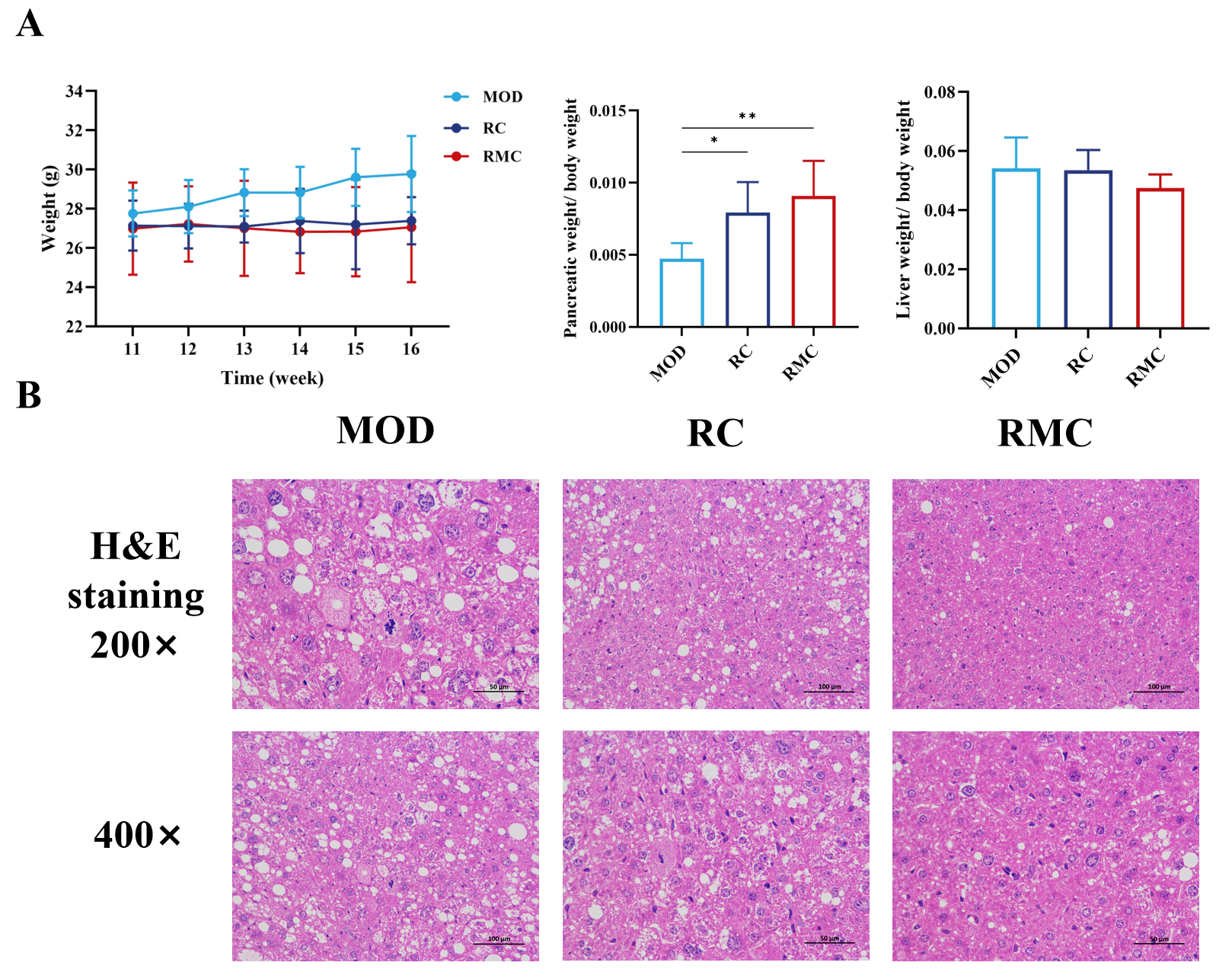

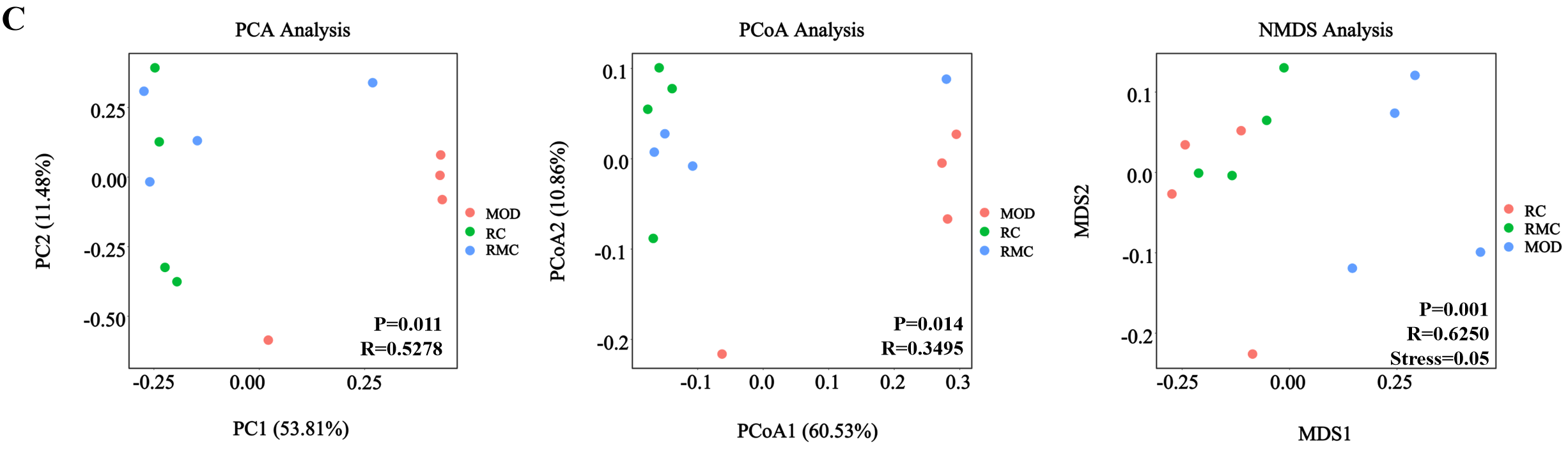


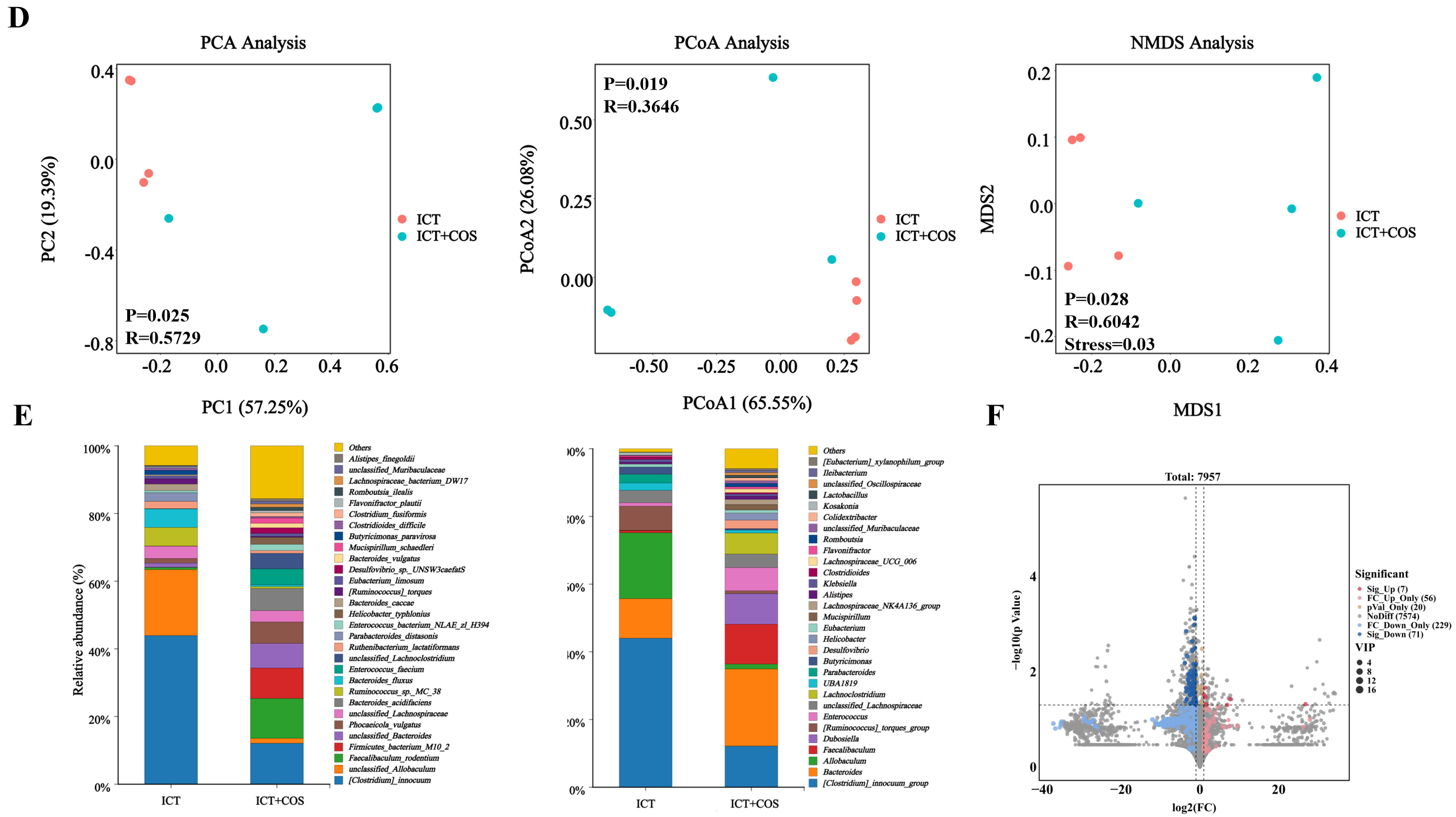


**Figure S3. Comprehensive impacts of COS intervention on mouse physiology, liver status and gut homeostasis.** (A) Body weight, pancreas-to-body weight ratio, and liver-to-body weight ratio of mice in each group. (B) H&E staining of the liver in mice from different treatment groups (Scale bar, 50 μm and 100 μm). (C-D) Beta diversity analysis of the gut bacterial community in mice from each group. (E) Histogram of gut bacterial distribution at the genus and species level. (F) Volcano plot analysis of fecal metabolites in ICT mice upon COS intervention. One-way ANOVA and Tukey's multiple comparisons test were utilized to compare multiple groups. Data are expressed as the mean ± SD, n = 6/group for (A), n = 4/group for (C-E); * *p* < 0.05, ** *p* < 0.01, and *** *p* < 0.001.
